# Total and whole grain intake in Latin America: findings from the multicenter cross-sectional Latin American Study of Health and Nutrition (ELANS)

**DOI:** 10.1007/s00394-021-02635-8

**Published:** 2021-07-07

**Authors:** Regina Mara Fisberg, Mariane Mello Fontanelli, Irina Kowalskys, Georgina Gómez, Attilio Rigotti, Lilia Yadira Cortés, Martha Yépez García, Rossina G. Pareja, Marianella Herrera-Cuenca, Mauro Fisberg, ELANS Study Group, Mauro Fisberg, Irina Kovalskys, Georgina Gómez Salas, Attilio Rigotti, Lilia Yadira Cortés Sanabria, Martha Cecilia Yépez García, Rossina Gabriella Pareja Torres, Marianella Herrera-Cuenca, Berthold Koletzko, Luis A. Moreno, Michael Pratt, Regina Mara Fisberg, Agatha Nogueira Previdelli, Viviana Guajardo, Ioná Zalcman Zimberg, Viviana Guajardo, María Paz Amigo, Ximena Janezic, Fernando Cardini, Myriam Echeverry, Natasha Aparecida Grande de França, Guadalupe Echeverría, Leslie Landaeta, Óscar Castillo, Luz Nayibe Vargas, Luisa Fernanda Tobar Yuri Milena Castillo, Rafael Monge Rojas, Mónica Villar Cáceres, María Belén Ocampo, María Reyna Liria, Krysty Meza, Mellisa Abad, Marianella Herrera- Cuenca, Maritza Landaeta-Jiménez, Betty Méndez, Maura Vásquez, Guillermo Ramírez, Pablo Hernández, Carmen Meza, Omaira Rivas, Vanessa Morales, Priscila Bezerra Gonçalves, Claudia Alberico, Gerson Luis de Moraes Ferrari

**Affiliations:** 1 Department of Nutrition, School of Public Health, University of São Paulo, Sao Paulo, 01246-904 Brazil; 2 Facultad de Ciencias Médicas, Carrera de Nutrición, Pontificia Universidad Católica Argentina, C1107 AAZ Buenos Aires, Argentina; 3 Departamento de Bioquímica, Escuela de Medicina, Universidad de Costa Rica, 11501-2060 San Jose, Costa Rica; 4 Centro de Nutrición Molecular y Enfermedades Crónicas, Departamento de Nutrición, Diabetes y Metabolismo, Escuela de Medicina, Pontificia Universidad Católica, 8330024 Santiago, Chile; 5 Departamento de Nutrición y Bioquímica, Pontificia Universidad Javeriana, 110231 Bogota, Colombia; 6 Colégio de Ciencias de la Salud, Universidad San Francisco de Quito, Quito, 17-1200-841 Ecuador; 7 Instituto de Investigación Nutricional, Lima, 15026 Peru; 8 Centro de Estudios del Desarrollo, Universidad Central de Venezuela (CENDES-UCV)/Fundación Bengoa, Caracas, 1053 Venezuela; 9 Fundação José Luiz Egydio Setubal, Instituto Pensi, Hospital Infantil Sabará, Sao Paulo, 01227-200 Brazil

**Keywords:** Latin America, Multicenter study, Epidemiology, Whole grain, Socioeconomic factors, Dietary intake

## Abstract

**Purpose:**

Understanding whole-grain intake and its associated factors is essential to tackle the double burden of malnutrition faced by Latin American countries. This study aimed to characterize total and whole grain intake in Latin American countries and to investigate foods contributing to these intake in the region.

**Methods:**

Data were obtained from the multicenter cross-sectional survey Latin American Study of Nutrition and Health (ELANS), including 9128 participants residing in urban areas of eight Latin American countries. Data collection was performed via two household visits using a standardized questionnaire and two 24 h dietary recalls. Usual dietary intake of total grain foods and foods containing whole grains was estimated. The association between the intake of grain food groups and sociodemographic variables was investigated using multiple linear regression models with random intercepts.

**Results:**

Mean intake of total grain foods and foods containing whole grains was 318.6 g/d and 14.7 g/d, respectively. Total grain foods were less consumed by participants at older ages (−9.8 g/d), and females (−9.9 g/d), and more consumed by those in the lowest socioeconomic category (24.8 g/d). Foods containing whole grains were more consumed by participants at older ages (3.3 g/d), and females (4.0 g/d), while those in the lowest socioeconomic category consumed 2.9 g/d less. Major contributors to energy provided from foods containing whole grains were oatmeal, masa *harina*, whole-wheat bread, corn chips, and wheat crackers.

**Conclusion:**

The intake of grain foods represented a substantial part of the Latin American population’s diet, but the intake of foods containing whole grains was extremely low in all assessed countries.

**Supplementary Information:**

The online version contains supplementary material available at 10.1007/s00394-021-02635-8.

## Introduction

Low whole grain (WG) intake is one of the main dietary risk factors contributing to deaths and disability-adjusted life-years (DALYs) globally, in particular, due to cardiovascular diseases and type 2 diabetes [1]. Inadequate WG intake is also linked to high economic burden, and even small increases in WG consumption may lead to health improvement as well as reduced health system burden, being considered a cost-saving strategy [2–4]. However, most nutrition surveillance surveys do not have WG intake estimations, since its assessment is still a challenging task. Variations in the definition of WG, labelling issues, and the lack of WG content in food composition tables make it difficult to estimate and compare WG intake between countries. Available data suggest that WG intake is far below recommended levels, especially among countries where grain foods are usually consumed as refined grains [3, 5]. Therefore, understanding WG intake as well as associated sociodemographic characteristics is essential to inform policymakers and stakeholders to reduce related disease burden.

In Latin American and the Caribbean countries, cardiovascular diseases and type 2 diabetes are the major causes of morbidity and mortality, and estimations from the Global Dietary Database Consortium indicate suboptimal diet is responsible for 53.8% of total cardiometabolic deaths in the region [6, 7]. Low WG intake, defined as the intake  < 125 g/d of foods containing  ≥ 1.0 g of fiber per 10 g of carbohydrate, occupied the fourth position among dietary risk factors, accounting for 9.2% of those deaths, more than 89 thousand deaths in 2010 [7]. Despite the overall low intake of whole grains in Latin American and Caribbean countries, the amount consumed varies widely (from 6 g/d in Cuba to 125 g/d in Barbados) [7]. Importantly, WG dietary sources were not previously investigated and may be culturally dependent. This knowledge is essential to encourage the population to increase the consumption of WG foods [3, 8]. Previous studies suggest that grain foods play a central role in the Latin American population’s diet, which makes them a potential target for policy actions to improve diet quality and to tackle the double burden of malnutrition in the region [9–11].

This study aims to provide data on WG intake in Latin American countries. The investigation was performed in three domains: (1) to estimate the intake of total and WG foods in Latin America, (2) to characterize this consumption by country, age, sex, and socioeconomic level; and (3) to assess foods contributing to grain intake in this region. To achieve these aims, we used the Estudio Latinoamericano de Nutrición y Salud (ELANS, Latin American Study of Nutrition and Health), a multicenter cross-sectional survey performed using a rigorous standardization protocol to harmonize food assessment and nutrient composition databases across participating countries generating comparable dietary intake data [12, 13].

## Methodology

### Study sample

ELANS was designed to collect dietary intake, physical activity and anthropometric data in a nationally representative urban sample of eight Latin American countries—Argentina, Brazil, Chile, Colombia, Costa Rica, Ecuador, Peru, and Venezuela [12], which accounted for approximately 63% of the Latin American population in 2015 according to the World Bank. Participants were randomly selected via complex multistage sampling. In the first stage, cities within the main urban regions of each country were selected, followed by census tracts. Systematic randomization process was used for household selection and participant’s inclusion was made using the following two criteria: the next birthday was used to select participants in half of the household sampled, and quotas of gender, age, and socioeconomic level were adopted to select participants in the other half of households. No more than one participant was included per household. Urban geographical location, sex, age (15–65 years) and socioeconomic level were considered as strata. A total of 10,134 participants were eligible for this study, but due to refusal, 9680 participants comprised the ELANS sample. Only those with complete data, who answered the socioeconomic questionnaire, and two 24 h dietary recalls were included in this study [*n* = 9128 participants from Argentina (*n* = 1266), Brazil (*n* = 2000), Chile (*n* = 879), Colombia (*n* = 1230), Costa Rica (*n* = 798), Ecuador (*n* = 800), Peru (*n* = 1113), and Venezuela (*n* = 1132)]. Data collection was performed via two household visits from September 2014 to August 2015 [12]. More information on the study design, protocol and methodology was previously published [12].

### Dietary intake

Dietary intake data were performed on non-consecutive days (within one week) via face-to-face interviews using two 24 h dietary recalls [13]. The five-step Multiple Pass Method was followed to enable complete and accurate 24 h recalls, and a photographic album of most consumed foods and commonly used household utensils assisted the portion sizes estimation [14]. Dietary data were converted into nutrients and other food components using the Nutrition Data System for Research software version 2014 developed by the Nutrition Coordinating Center, University of Minnesota, Minneapolis, MN, which uses the United States Department of Agriculture (USDA) food composition table as the main data source. Therefore, nutritional values of foods included in the survey were compared with the ones available in the local food composition tables and a concordance rate between 80 and 120% for energy and macronutrient content was required for the food item to be selected. Regional foods and recipes were also inserted in the program based on local food composition tables and local publications. Plausibility checks were performed with the purpose of identifying and correcting possible errors in dietary data collection and processing. Kovalskys et al. [13] described more details about the dietary collection and standardization process.

### Grain food products

The USDA “grain products” food group was used to identify grain foods since it has a direct link to food items in the NDSR database. This food grouping system includes the following subcategories in the “grain products” group: (1) flour and dry mixes, (2) yeast breads, rolls, (3) quick breads, (4) cakes, cookies, pies, pastries, bars, (5) crackers and salty snacks from grain products, (6) pancakes, waffles, French toast, other grain products, (7) pastas, cooked cereals, rice, (8) cereals, not cooked or not specified as to cooked, (9) grain mixtures, frozen plate meals, soups (10) meat substitutes, mainly cereal protein [15]. Local grain foods inserted in the program were manually assigned into one of the subcategories based on nutritional similarity and culinary use. Foods consumed at the first and second 24 h recalls were evaluated with mixed dishes disaggregated.

Grain products were categorized according to the whole grain content in the following groups: (1) total grain foods, (2) grain foods containing WG (any amount), and (3) grain foods containing  > 50% WG. The third category is included in the second, and both are comprised in the first category. The classification was made using the “total grain (ounce equivalent)” and “whole grain (ounce equivalent)” variables of the NDSR software (see Supplementary Materials—Online Resource Table 1). We opted to work on the food basis since there is no information on WG content in Latin American food composition tables or any other dataset of our knowledge that would enable us to estimate the WG content of foods in grams on a dry weight basis as previously recommended [16]. Therefore, a plausibility check for foods consumed among ELANS participants was only possible by using the presence and position of a WG ingredient in the ingredient list on the food label of products commercialized in Latin American countries.

Each individual’s usual intake of energy (kcal), total grain foods (g), grain foods containing WG (g), and grain foods containing  > 50% WG (g) was estimated using the National Cancer Institute (NCI) method by MIXTRAN and INDIVINT SAS macros [17]. A two-part model was considered for grain foods containing WG and grain foods containing  > 50% WG (more than 5% of zero intake), accounting for the probability to consume food and the amount of food consumed. For total grain foods and total energy, only the “amount” model was performed. For both probability and amount models, covariates included age, sex, and an indicator of first-day versus second-day dietary recall to account for sequence effects of a subject’s dietary recall. The estimation of usual intake was conducted separately for each country to account for differences in food intake among studied countries.

The usual intake of grain foods was further adjusted for total energy intake to grams per 2000 kcal/d using the nutrient density method to consider variations in body size, physical activity and metabolic efficiency [18]. We used 2000 kcal for this adjustment as this value is similar to the mean energy intake among ELANS participants [mean 1997.7 kcal/d, standard deviation (SD) 505.9 kcal/d].

### Sociodemographic variables

Sociodemographic variables were assessed using a standardized questionnaire during the first household visit. Age (15–19 years, 20–34 years, 35–49 years, 50–65 years), sex (male, female), and socioeconomic level (high, middle, low) were used in this study. Socioeconomic level varied according to the country, based on the legislative requirements and local standard layouts, and was categorized into three levels based on national indexes [12].

### Statistical analysis

The absolute and relative number of grain food products according to grain food groups and countries were computed. Energy-adjusted mean (SD) and median (interquartile range, IQR) intake of grain foods were evaluated according to countries and sociodemographic variables. The association between the intake of grain food groups and sociodemographic variables was investigated using multiple linear regression models with random intercepts to account for the correlation between observations within urban centers included in the study. Models were adjusted for age group (15–19 years, 20–34 years, 35–49 years, 50–65 years), sex (male, female), socioeconomic level (high, medium, low), and center (urban centers included in ELANS, random intercept). Coefficients from these models were used to estimate adjusted mean and median intakes, and the proportion of foods containing WG to total grain foods consumed.

To assess which foods contribute to energy intake from grain food groups among Latin American population, we estimated the corresponding percentage of energy from total grain foods, foods containing WG, and foods containing  > 50% WG from the total energy provided from these foods. For this analysis, the mean energy intake from grain food groups between the first and second 24 h recall was used.

Data were analyzed using Stata version 14.0 (StataCorp) and statistical significance was set as two-sided *p* < 0.05.

## Results

### Number of grain food products in the ELANS dataset

**Fig. 1:**
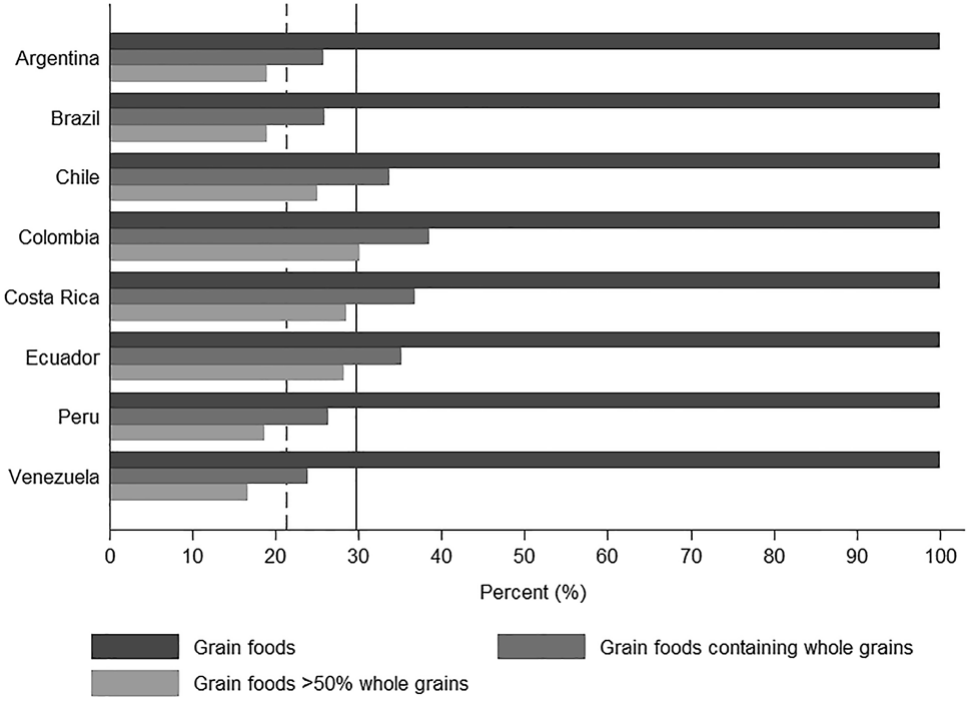
Percent of total grain foods, grain foods containing whole grains and grain foods containing  > 50% whole grains in the Latin American Study of Nutrition and Health (ELANS) database according to country, 2015. Black solid line represents the proportion of foods containing whole grain in ELANS database. Black dashed line represents the proportion of foods containing  > 50% whole grain in ELANS database

A total of 695 grain food products were identified in ELANS dataset, including 206 (29.6%) grain foods containing WG, and 148 (21.3%) grain foods containing  > 50% WG. The number of products varied between countries, the highest number of total grain foods was seen in Argentina (*n* = 217) followed by Colombia (*n* = 213), Costa Rica (*n* = 193), Brazil (*n* = 185), Chile (*n* = 172), Peru (*n* = 156), Ecuador (*n* = 145), and Venezuela (*n* = 126). The percent of grain products containing WG from total grain products was higher in Colombia (38.5%, *n* = 82), followed by Costa Rica (36.8%, *n* = 71), Ecuador (35.2%, *n* = 51), Chile (33.7%, *n* = 58,), Peru (26.3%, *n* = 41), Brazil (25.9%, *n* = 48), Argentina (25.8%, *n* = 56), and Venezuela (23.8%, *n* = 30). The percent of grain foods containing  > 50% WG from total grain products followed a similar pattern: Colombia (30.0%, *n* = 64), Costa Rica (28.5%, *n* = 55), Ecuador (28.3%, *n* = 41), Chile (25%, *n* = 43), Argentina (18.9%, *n* = 41), Brazil (18.9%, *n* = 35), Peru (18.6%, *n* = 29), and Venezuela (16.7%, *n* = 21) (Fig. 1 and Online Resource Table 2) [1].

### Intake of grain foods by the studied Latin American population

**Fig. 2:**
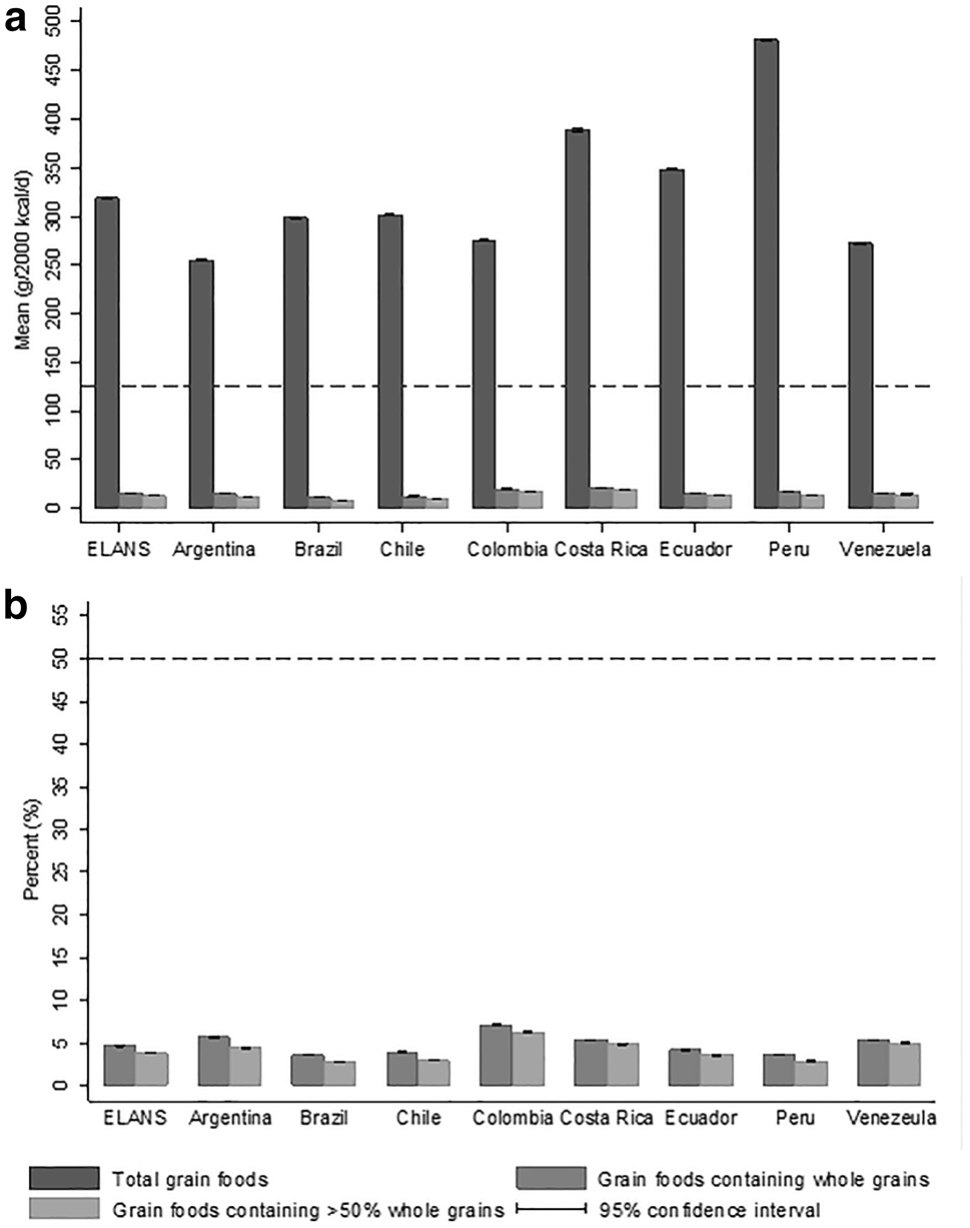
Intake of grain foods in Latin American countries according to whole grain content based on the Latin American Study of Nutrition and Health (ELANS), 2015. **A** Mean intake (g/2000 kcal/d), **B** Proportion foods containing whole grain and foods  > 50% whole grain to total grain foods. Black dashed line represents the recommended intake. Estimates obtained from linear regression models adjusted for age group, sex, socioeconomic level and study center (random effect)

Mean (SD) intake of total grain foods in ELANS was 318.6 g/d (9.6), and ranged from 254.8 g/d (12.6) in Argentina to 481.7 g/d (25.2) in Peru. Mean (SD) intake of foods containing WG and foods containing  > 50% WG was 14.7 g/d (2.6) and 12.2 g/d (1.8), respectively. Brazil had the lowest mean intake of both grain food groups containing WG, 10.6 g/d (SD 2.3) for grain foods containing WG and 8.2 g/d (SD 1.6) for grain foods containing  > 50% WG, while Costa Rica presented the highest intakes, 20.8 g/d (SD 2.8) and 18.9 g/d (SD 2.1), respectively (Fig. 2 and Online Resource Table 3) [1].

The estimated proportion of foods containing WG to total grain foods consumed was 4.6% (SD 0.9) and for foods containing  > 50% WG, this percentage was 3.9% (SD 0.7). Colombia presented the highest proportions, 7.1% (SD 1.4) for grain foods containing WG, and 6.3% (SD 1.0) for grain foods containing  > 50% WG. The lowest proportion was seen in Brazil [3.6% (SD 0.9) for grain foods containing WG, and 2.8% (SD 0.6) for grain foods containing  > 50% WG], and in Peru [3.6% (SD 0.5) for grain foods containing WG and 2.8% (SD 0.4) for grain foods containing  > 50% WG] (Fig. 2 and Online Resource Table 4) [1].

### Sociodemographic differences in grain foods intake in Latin America

**Fig. 3:**
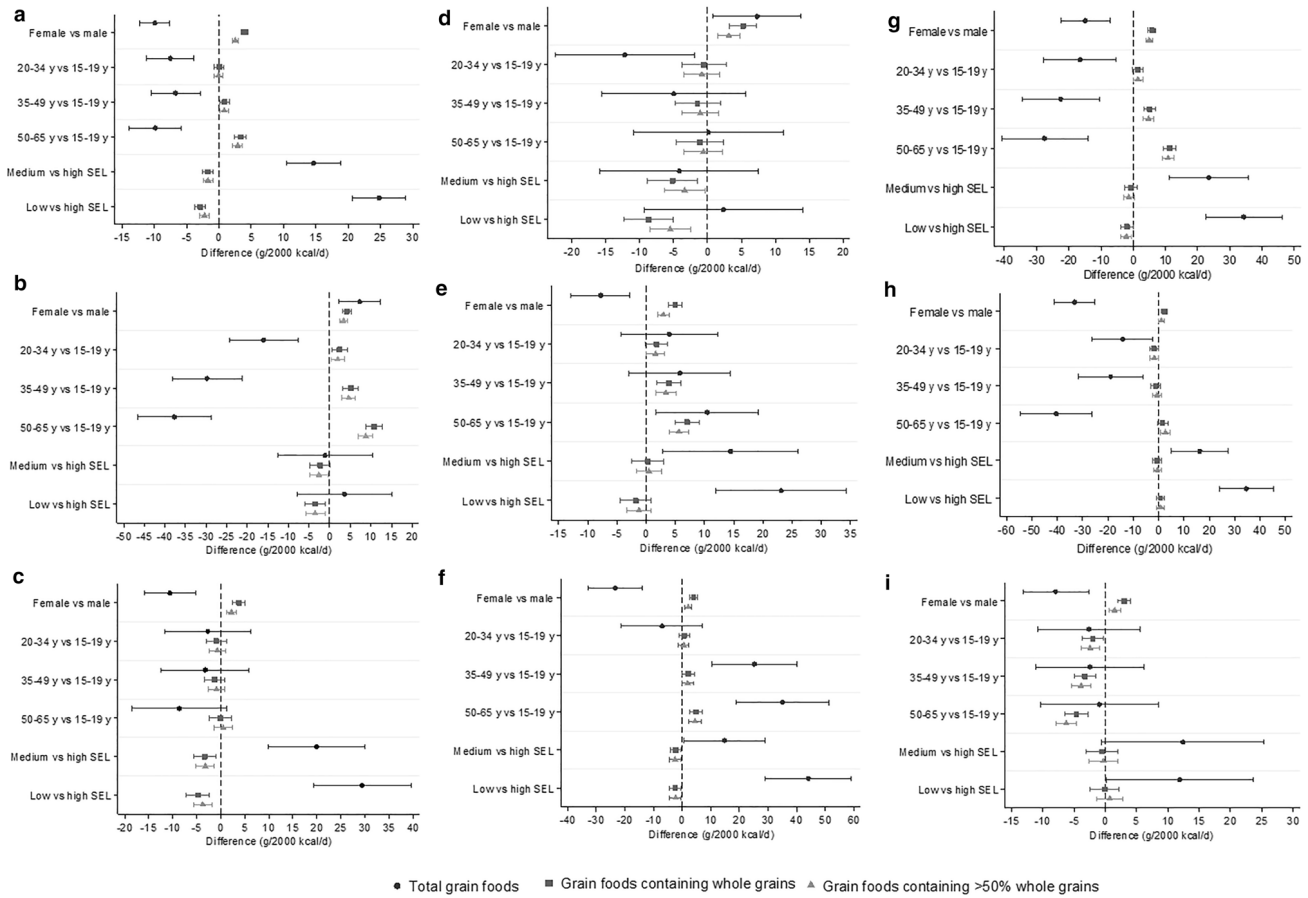
Sociodemographic variables associated with grain foods intake in Latin American countries based on the Latin American Study of Nutrition and Health (ELANS). *y* years, *SEL* socioeconomic level, *vs* versus. Letters indicate: **a** ELANS (*n* = 9218), **b** Argentina (*n* = 1266), **c** Brazil (*n* = 2000), **d** Chile (*n* = 879), **e** Colombia (*n* = 1230), **f** Costa Rica (*n* = 798), **g** Ecuador (*n* = 800), **h** Peru (*n* = 1113), (i) Venezuela (*n* = 1132). Error bars represent 95% confidence interval. Estimates obtained from linear regression models with random intercept for study center

The association between sociodemographic variables and the intake of total grain foods, foods containing WG, and foods containing > 50% WG in assessed countries is presented in Fig. 3 and Online Resource Table 5 [1]. Regarding the consumption of total grain foods, participants aged 20–34 years, 35–49 years, and 50–65 years consumed 7.5 g/d (95% CI 11.2, 3.9), 6.7 g/d (95% CI 10.5, 2.9), and 9.8 g/d (95% CI 13.9, 5.8) less when compared to those aged 12–19 years, respectively, women consumed 9.9 g/d (95% CI 12.2, 7.6) less than men, and those in the medium and low socioeconomic levels consumed 14.7 g/d (95% CI 10.5, 18.9) and 24.8 g/d (95% CI 20.7, 29.0) more than those in the high socioeconomic level category, respectively.

For foods containing WG, participants aged 35–49 years and 50–69 years consumed 0.9 g/d (95% CI 0.1, 1.7) and 3.3 g/d (95% CI 2.5, 4.1) more than those aged 15–19 years, respectively; women consumed 4.0 g/d (95% CI 3.5, 4.5) more than men; and participants at medium and low socioeconomic levels consumed 1.73 g/d (95% CI 2.6, 0.9) and 2.9 g/d (95% CI 3.7, 2.0) less than those at high socioeconomic level, respectively. For foods containing  > 50% WG, the pattern was similar to the one observed for grain foods containing WG. Participants aged 35–49 years and 50–65 years consumed more from this food group than participants aged 15–19 years [0.8 g/d (95% CI 0.2, 1.5) and 2.9 g/d (95% CI 2.2, 3.6), respectively], females consumed 2.5 g/d (95% CI 2.1, 2.9) more than males, and participants categorized in the medium and low socioeconomic level consumed less grain foods containing  > 50% WG than those in the high socioeconomic level [1.7 g/d (95% CI 2.4, 1.0) and 2.2 g/d (95% CI 3.0, 1.5), respectively].

Generally, a similar scenario was seen across countries. However, differences were observed for total grain foods in Argentina and Chile, where women consumed more total grain foods than men [7.3 g/d (95% CI 2.4, 12.3) in Argentina and 7.3 g/d (95% CI 0.8, 13.7) in Chile]. Another difference was the higher consumption of grain foods by older participants when compared to those at younger ages in Colombia [10.4 g/d (95% CI 1.7, 19.2) for 50–65 years vs.15–19 years] and Costa Rica [25.3 g/d (95% CI 10.4, 40.1) for 35–49 years vs. 15–19 years, and 35.2 g/d (95% CI 19.1, 51.3) for 50–65 years vs. 15–19 years].

### Foods contributing to energy intake from grain foods in Latin America

White rice (22.3%), French bread (9.2%), white flour (7.4%), spaghetti noodles (5.5%), and baguette (4.6%) were the main food items contributing to energy provided from grain foods in Latin American diet, accounting for approximately 50% of energy provided from these foods. The main contributors to foods containing WG and foods containing  > 50% WG were nearly the same and included the following foods: oatmeal (11.6%), masa harina (11.0%), whole-wheat bread (6.6%), corn chips (4.8%), and wheat crackers (4.1%). These food items accounted for approximately 40% of energy provided from these foods. Grain foods varied across countries, but the low number of participants consuming foods containing WG was consistent in all the studied countries (Table 1).

**Table 1:** Foods contributing to energy intake from total grain foods, foods containing whole grain, and foods containing  > 50% whole grains among population residing in Latin America based on the Latin American Study of Nutrition and Health, 2015

Country	Total grains				Foods containing whole grains				Foods containing > 50% whole grains			
	Foods	*n*	%	∑	Foods	*n*	%	∑	Foods	*n*	%	∑
ELANS	Rice, white, regular cooking	7580	22.3	22.3	Oatmeal, dry	777	11.6	11.6	Oatmeal, dry	777	13.9	13.9
	French bread	3059	9.2	31.5	Corn flour, masa harina	1544	11.0	22.6	Corn flour, masa harina	1544	13.2	27.1
	Flour, white all-purpose, enriched	3994	7.4	38.9	Whole wheat bread, regular, commercial	295	6.6	29.2	Whole wheat bread, regular, commercial	295	7.9	35.0
	Spaghetti noodles, white	1922	5.5	44.3	Corn chips, ingredient fat not known	102	4.8	33.9	Corn chips, ingredient fat not known	102	5.7	40.8
	Bread, loaf, baguette	1436	4.6	49.0	Crackers, wheat, regular	155	4.1	38.0	Whole wheat bread, regular, homemade or bakery	71	3.1	43.9
Argentina	Bread, loaf, baguette	872	17.4	17.4	Whole wheat bread, regular, commercial	53	12.1	12.1	Whole wheat bread, regular, commercial	53	17.8	17.8
	Flour, white all-purpose, enriched	1003	14.0	31.4	Whole wheat bread, regular, homemade or bakery	33	10.7	22.8	Whole wheat bread, regular, homemade or bakery	33	15.8	33.6
	Noodles, white (egg-type)	418	6.6	37.9	Crackers, whole wheat, regular	54	10.2	33.0	Crackers, whole wheat, regular, regular	54	15.0	48.6
	*Libritos o criollitos* (bizcochitos)	235	5.6	43.5	Crackers, wheat, regular	41	9.2	42.1	Noodles, whole wheat	78	11.3	59.9
	Grains, rice, white, regular cooking	439	4.8	48.4	Crackers, saltine or soda, whole wheat	42	8.2	50.3	Crackers, rice cake, plain, regular	26	4.1	64.0
Brazil	Bread, loaf, French bread	1466	21.7	21.7	Crackers, wheat, regular	103	19.6	19.6	Corn chips, ingredient fat not known	13	12.5	12.5
	Grains, rice, white, regular cooking	1862	20.3	42.0	Corn chips, ingredient fat not known	13	9.3	29.0	Popcorn, microwave popped from package, regular	15	12.4	24.9
	Spaghetti noodles, white	616	9.2	51.1	Popcorn, microwave popped from package, regular	15	9.3	38.3	Whole wheat bread, regular, commercial	65	11.6	36.5
	Flour, white all-purpose, enriched	696	5.7	56.8	Whole wheat bread, regular, commercial	65	8.6	46.9	Crackers, whole wheat, reduced fat	42	9.9	46.4
	Crackers, regular	396	4.7	61.6	Crackers, whole wheat, reduced fat	42	7.4	54.3	Spaghetti noodles, whole wheat	28	7.3	53.7
Chile	French bread	660	31.7	31.7	Whole wheat bread, regular, commercial	38	18.1	18.1	Whole wheat bread, regular, commercial	38	23.7	23.7
	Hallulla (bread)	376	19.5	51.1	Popcorn, caramel or sugar coated, commercially prepared	5	7.8	25.9	Popcorn, caramel or sugar coated, commercially prepared	5	10.2	33.9
	Grains, rice, white, regular cooking	526	10.0	61.2	Cookies and bars, oatmeal, commercial package, regular	11	7.7	33.6	Oatmeal, dry	38	8.8	42.7
	Spaghetti noodles, white	204	6.5	67.7	Cookies and bars, graham cracker, plain	25	6.9	40.5	Snacks, granola bars, cereal bar, regular	23	7.2	49.9
	Flour, white all-purpose, enriched	291	5.4	73.1	Instant oatmeal (fortified), plain	38	6.7	47.2	Cereal, ready-to-eat, General Mills, Wheaties	8	6.3	56.1
Colombia	Grains, rice, white, regular cooking	1180	25.9	25.9	Corn flour, masa harina	474	36.2	36.2	Corn flour, masa harina	474	40.4	40.4
	White bread, regular, commercial	581	11.4	37.3	Oatmeal, dry—unprepared, steel cut	49	8.1	44.3	Oatmeal, dry—unprepared, steel cut	49	9.0	49.5
	Flour, white all-purpose, enriched	732	10.8	48.1	Oatmeal—dry, regular or quick	50	5.4	49.7	Ingredient, oatmeal—dry, regular or quick	50	6.1	55.5
	Bread, rolls, white, baked commercially	172	5.2	53.3	Whole wheat bread, regular, commercial	27	5.1	54.8	Whole wheat bread, regular, commercial	27	5.7	61.2
	Corn flour, masa harina	474	4.8	58.1	Corn chips, ingredient fat not known	27	3.8	58.6	Corn chips, ingredient fat not known	27	4.2	65.4
Costa Rica	Grains, rice, white, regular cooking,	731	30.3	30.3	Oatmeal, dry—unprepared, regular or quick	80	23.1	23.1	Oatmeal, dry—unprepared, regular or quick	80	25.3	25.3
	Fortified bread	557	12.4	42.6	Tortilla maiz fortified	128	13.2	36.2	Tortilla maiz fortified	128	14.4	39.7
	Arroz cocido fortificado	193	6.4	49.0	Corn chips, ingredient fat not known	24	12.6	48.9	Corn chips, ingredient fat not known	24	13.8	53.5
	Flour, white all-purpose, enriched	259	3.8	52.8	Whole wheat bread, regular, commercial	33	4.4	53.3	Whole wheat bread, regular, commercial	33	4.9	58.4
	Flour, corn, masa, white—unenriched	73	3.1	55.9	Popcorn, microwave popped from package, regular	11	4.0	57.3	Popcorn, microwave popped from package, regular	11	4.4	62.8
Ecuador	Grains, rice, white, regular cooking,	789	38.6	38.6	Oatmeal—dry, regular or quick	125	17.2	17.2	Oatmeal—dry, regular or quick	125	20.1	20.1
	Rolls, white, baked from recipe or bakery	457	16.9	55.5	Bread, rolls, whole wheat	31	16.4	33.7	Bread, rolls, whole wheat	31	19.2	39.3
	Flour, white all-purpose, enriched	307	7.5	63.0	Cookies and bars, graham cracker, plain	26	10.3	43.9	Whole wheat bread, regular, homemade or bakery	21	10.7	49.9
	White bread, regular, homemade or bakery	192	5.2	68.2	Whole wheat bread, regular, homemade or bakery	21	9.1	53.1	Corn chips, ingredient fat not known	18	8.8	58.7
	Pasta, fideos, white	172	3.2	71.4	Corn chips, ingredient fat not known	18	7.5	60.6	Quinoa, dry	18	5.0	63.7
Peru	Grains, rice, white, regular cooking	1085	41.9	41.9	Oatmeal—dry, regular or quick	400	28.1	28.1	Oatmeal—dry, regular or quick	400	34.8	34.8
	French bread	719	11.1	53.1	Quinoa, dry	128	9.0	37.1	Quinoa, dry	128	11.2	46.0
	Spaghetti noodles, white	558	10.9	63.9	Ingredient, bulgur—dry	65	8.4	45.5	Bulgur—dry	65	10.5	56.5
	Flour, white all-purpose, enriched	342	3.4	67.4	Wheat bread, regular	50	8.4	53.9	Whole wheat bread, regular, commercial	56	8.0	64.5
	Bread, rolls, white, baked commercially	196	3.4	70.8	Whole wheat bread, regular, commercial	56	6.5	60.3	Snacks, popcorn, home popped, hot air popped	34	6.8	71.3
Venezuela	Flour, corn	1041	22.3	22.3	Flour, corn, masa harina	240	29.9	29.9	Flour, corn, masa harina	240	31.6	31.6
	Grains, rice, white, regular cooking	968	14.7	37.0	Flour, white whole wheat	131	14.8	44.7	Flour, white whole wheat	131	15.6	47.2
	Bread, loaf, baguette	505	13.5	50.5	Oatmeal—dry, regular or quick	91	12.6	57.3	Oatmeal—dry, regular or quick	91	13.3	60.5
	Pasta, macaroni noodles, white	207	7.1	57.6	Flour, corn, whole grain, white	35	11.4	68.7	Flour, corn, whole grain, white	35	12.1	72.6
	Flour, white all-purpose, enriched	364	6.4	64.0	Chips—snack type, taco or tortilla	23	7.0	75.8	Chips—snack type, taco or tortilla	23	7.4	80.0

*n* number of participants who consumed the respective food item, % percent of energy from total grain foods and whole grain foods (according to whole grain content), ∑ cumulative percent of energy

## Discussion

In this multicenter cross-sectional study including eight Latin American countries compared using a standardized dietary database, the intake of total grain foods represented a substantial part of the population’s diet (mean intake of 318 g/d or  > 10.5 servings, considering 30 g as a standard serving of a grain food product) [19]. However, the intake of grain foods containing WG was extremely low in all assessed countries. Less than 5% of total grain foods intake (14.7 g/d, < 0.5 serving) came from foods containing WG. Overall, participants at younger ages, males, and those at lower socioeconomic levels consumed more total grain foods, while an inverse pattern was seen for foods containing WG, which were more consumed by participants at older ages, females, and those at higher socioeconomic levels. Our findings indicate that public health actions to increase WG intake are urgently needed in Latin American countries.

Several grain foods stand for Latin American typical foods, mostly consumed in their refined version at the present time. Grains are the most recommended food group by Latin American food-based dietary guidelines, and are also included in the guidelines’ visual representations of many countries [5, 20]. Fried corn tortillas, burritos, and tacos, for example, are illustrated in the Chilean, Mexican, Paraguayan, and Uruguayan visual representations, while quinoa and amaranth were represented in the Bolivian Arco de la Alimentación [20–25]. Most guidelines encourage the consumption of grain foods in their WG form; however, WG recommendations remain predominantly qualitative, making it difficult for the population to be aware of the amount of WG needed to achieve health benefits [3, 26]. In addition, WG intake is the result of grains (e.g. oatmeal and quinoa) and grain-based products (e.g. whole wheat bread and whole wheat crackers), which contain a mix of different grains and other ingredients, and despite efforts made to establish a global consensus on the amount of WG a food must contain to be labeled as WG, this definition depends on each country legislation [19, 27].

Global intake of WG (defined as foods containing  ≥ 1.0 g of fiber per 10 g of carbohydrate) was estimated to be 29 g/d in 2017 by the Global Burden Disease Study, 23% of the optimal level [1]. In 2011–2012, WG-food intake was 30 g/d among United States of America adults [28]. In Europe, the intake of WG, defined as an ingredient in WG-containing foods, ranged from 14.4 g/d in France to 58 g/d in Sweden among adults, data collected in 2010 and 2000, respectively [3, 29, 30]. Kissock et al. estimated the WG intake of Australian adults to be 32.6 g/d in 2011–2012 by using the Healthgrain Forum WG food definition [31]. Our results indicate that the intake of foods containing WG in Latin American countries was approximately 15 g/d in 2015. Notably, comparison of WG intake among countries is limited due to several reasons. First, the different periods in which the evaluations were carried out correspond to varying availability of WG food products to the studied population [32]. Second, the methods used to assess food consumption will possibly impact WG estimates, food frequency questionnaires, for example, may not contain all the WG foods available to the population [32]. Third, in most countries food composition tables do not contain the WG content of food items, which difficult accurate estimates [3, 32]. Finally, the adopted WG definition impact intake estimates and varies across studies [3, 31, 32]. Despite the limitations and regardless of the definition used, it is possible to infer that the consumption of WG is below recommended levels in most countries, especially in the Latin America region.

One aspect required to improve WG intake in Latin America is to make WG foods more accessible. The intake of WG was previously related to dietary inequalities and evidence points to the higher price of these foods when compared to equivalent refined options [28, 33–36]. Our findings corroborate with the previous literature, given that participants at lower socioeconomic level consumed less WG foods compared to those at higher socioeconomic levels (−3 g/d). However, this association was not significant in all studied countries possibly due to the small amounts of WG-containing foods consumed, and due to the food items contributing to WG intake. Masa harina, for example, can be considered a staple food in many Latin American countries and was the main contributor to WG intake in Colombia, where differences between socioeconomic levels were not found. In this same country, prior study reported that participants in the highest socioeconomic level had the worst diet quality among Colombian children and adults, and WG was the category with the lowest contribution to Alternative Health Eating Index (AHEI) score in this population (0.4 points in 2005 and 0.2 points in 2015 from 10 points total) [37].

Beyond WG, higher consumption of refined grain foods among lower socioeconomic levels deserves further attention. From 1999 to 2016, reduced intake of low-quality carbohydrate, characterized by refined grains, potato, starchy vegetables other than potatoes, added sugars, and 100% fruit juices, were greater among the highest level of income (− 3.9% of energy) when compared to the lowest income level (− 2.5% of energy) among United States of America adults [38]. Disparities were also seen among youth, whose intake of refined grains significantly increased among Mexican American (from 6.2 to 6.6 servings/d) but remained stable among non-Hispanic white and non-Hispanic black in the same period of time [39]. In the present study, a difference of 24.8 g/d (~ 0.8 servings) was seen when participants in the lower socioeconomic level were compared to those in the higher socioeconomic level, reinforcing the key role of policies to improve Latin Americans diet quality, ensuring that low-income subgroups benefit from these actions.

Because individuals at younger ages and males usually present poorer diet quality, the lower intake of foods containing WG by these population subgroups was expected and previously reported [33, 35, 40]. Although evidence points to some improvement along with ageing, starting the population to eat WG at younger ages seems to be an effective strategy for a lifelong dietary pattern that includes WG foods [41, 42]. Despite the observed differences, the intake of foods containing WG was very low among all studied countries, suggesting that even small increases in WG intake may promote substantial health benefits and probably reduce the burden of healthcare systems [3, 4]. Currently, Latin American countries face a challenging scenario, where economic crisis, including the stagnation of the reducing poverty and inequality trends, effects of climate change, food insecurity and the double burden of malnutrition coexist [43]. The inclusion of WG foods in already existing policies and the formulation of new actions aiming at increasing WG intake are required to move toward an adequate and sustainable diet in the region [44].

This study has several limitations. First, we were not able to quantify the WG content of foods in grams on a dry weight basis as previously recommended [16]. The main nutrient data source in this study was the USDA food composition table, and despite the corrections made to account for the nutrient content of foods consumed by the studied population, there is no information on WG content of these foods available in Latin American food composition tables [13]. Masa harina, for example, is considered whole grain by the USDA given the process of treating the whole corn with lime, which increases the bioavailability of B vitamins and resembles the whole corn flour nutrient content [45, 46]. This food item contributes to fiber intake in Latin American countries; however, the extent of processing losses related to parts of the grain was not locally evaluated [11]. In this sense, we opted to categorize grain foods as containing WG and containing  > 50% WG, so that a plausibility check could be performed based on the ingredient list on the food label. Therefore, estimations provided in this study are probably overestimated. To overcome this key limitation, efforts to develop a WG database for foods consumed in the region are needed. In Australia, for example, a food composition table including the WG content of foods was developed using a recipe-based method, with contribution from industry stakeholders, product packaging, and ingredient list and it is now used to estimate WG intake in the country [31, 47]. Second, dietary intake data were based on self-reported 24 h recalls, and it is susceptible to random and systematic errors. Errors were minimized by collecting the second 24 h dietary recall, applying the National Cancer Institute method to estimate the usual intake, and further considering energy intake for estimates [17, 18]. In addition, 24 h recalls were collected by trained interviewers using standardized methods, such as the multiple pass method and a uniform procedure to detail reported food items [13, 14]. Third, food frequency questionnaires were not collected and the distinction between real WG consumers from non-consumers of WG foods was not possible [18]. Fourth, ELANS included participants living in urban areas of each country, and although 80–90% of Latin American populations live in urban areas, findings cannot be extrapolated to the entire country population [12]. In addition, ELANS did not include all Latin American countries and results may not be generalizable to other countries in the region [12]. Future surveys should comprise a great number of Latin American countries and include the rural population.

Strengths of the current study include the comprehensive analyses of the intake of total grains, foods containing WG, and foods containing  > 50% WG using a standardized dietary database [13]. Importantly, this investigation used data from eight Latin American countries, where the low intake of WG is among the leading dietary risk factors responsible for deaths and DALYs [1, 7]. We further advance by characterizing sociodemographic characteristics associated with total grain and WG-containing foods intake, and investigated foods contributing to this consumption. Results provide evidence for future sociodemographic-targeted and culturally specific interventions aiming to improve diet quality in the region.

The discussion of what constitutes a WG food is beginning to emerge in Latin American countries and it will be a crucial step to improve WG intake levels in the region. In Brazil, for example, the proposed definition established that a product must contain at least 30% of WG ingredients, and a greater amount of WG than the refined grain ingredients. This legislation may enter into force soon [48]. However, in addition to WG content, it is recommended that the food item meet a locally accepted healthy nutrition criterion to be labeled as WG [19], and this is essential to improve population’s nutrition by encouraging them to increase WG intake. Previous study in Brazil that evaluated breads, biscuits and toasts labeled as WG available in supermarkets, found that 64.6% (from the 147 assessed products) did not present WG flour as the first ingredient, 53.7% presented excessive levels of sodium, and 22.4 and 56.5% had excessive levels of total and saturated fat, respectively [49]. Likewise, after the exclusion of discretionary foods from WG estimations in the Australian National Nutrition and Physical Activity Survey, WG intake values decreased by approximately 1–5 g/d, especially among older children and adolescents [31]. Results from studies investigating how carbohydrate-rich foods meet nutrient profiling system and different ratios between carbohydrate, fiber, and free sugar also endorsed the importance of evaluating the nutrient content of these foods, which comprise a great variety of products [50, 51]. We opted not to use a healthiness criterion to exclude grain foods containing WG in our analysis since the amount consumed was already too small, but it would be essential for labeling purposes to help consumers to make the best possible choices (i.e. healthier and higher WG content products).

Despite the different definitions used to characterize WG in studies, low WG intake is commonly observed among Latin American populations [7, 33, 37, 52], and optimal intake of WG was previously found in 2.4% of Latin American populations [53]. The intake of foods containing WG in ELANS was only 15 g/d, and slightly less for foods containing  > 50% WG (12 g/d). Costa Rica presented the highest consumption (~ 21 g/d) while Brazil had the lowest intake of foods containing WG (~ 11 g/d), but the amount consumed by all countries was far below recommended levels. At the same time, there was a high intake of total grain foods, and this gap between WG and total grains consumption may represent an opportunity for public health actions aiming at increasing WG intake in Latin American populations. Additionally, our results indicate that countries with the highest WG intake also had a greater variety of food items containing WG (Colombia and Costa Rica), suggesting it is also important to increase the availability of WG food options in Latin America.

Successful approaches to promoting whole grain consumption are still scarce, but are increasing around the world [8, 42]. Initiatives from countries such as Denmark, Philippines, Singapore, and the United States were recently described and could be adapted to Latin American populations [42]. Particularly the campaign from Philippines, which encouraged consumers to switch from white to brown rice by means of social responsibility appeal and financial incentives for selling and purchasing brown rice [54]. This case is an important reference to Latin American countries given the focused efforts on rice, which was the major contributor to energy intake provided from grain foods among the studied countries (more than 20% of total energy), and was among the top five contributors to grain foods in all participating countries. In addition to white rice, common food items contributing to total grain food intake included white flour, noodles, corn flour, different types of breads and some typical grain-based foods (*Libritos o criollitos* in Argentina and Hallulla in Chile). Each country had its own specificities, but common food items contributing to total grain consumption may represent a vehicle for increasing WG intake among Latin American populations.

In conclusion, the intake of grain foods represented a substantial part of the Latin American population’s diet, but the intake of grain foods containing WG was extremely low in all assessed countries. Generally, participants at younger ages, males, and those at lower socioeconomic level consumed more total grain foods, while an inverse pattern was seen for foods containing WG, which were more consumed by participants at older ages, females, and those at higher socioeconomic levels. These findings provide support for subsequent interventions and policy actions aiming at increasing WG intake among Latin American populations.

## Supplementary Information

Below is the link to the electronic supplementary material.

Supplementary file1 (DOCX 52 KB)

## Data Availability

Due to ethical and legal restrictions of the eight institutions involved, the data underlying this study are available upon request and must be approved by the Publishing Committee of ELANS. Data are available for researchers who meet the criteria for access to confidential data and also approved by the Publishing Committee of ELANS. To apply for access to these data, interested researchers must submit a detailed project proposal to ELANS. The authors confirm that the data underlying this study will be shared provided that requests are submitted through appropriate channels via the email: mauro.fisberg@pensi.org.br.

## Code availability

The code used in this study is available upon request.

## References

[1] GBD (2017). Diet collaborators (2019) health effects of dietary risks in 195 countries, 1990–2017: a systematic analysis for the Global Burden of Disease Study 2017. Lancet.

[2] Lieffers JRL, Ekwaru JP, Ohinmaa A, Veugelers PJ (2018). The economic burden of not meeting food recommendations in Canada: The cost of doing nothing. PLoS ONE.

[3] Miller KB (2020). Review of whole grain and dietary fiber recommendations and intake levels in different countries. Nutr Rev.

[4] Hu Y, Ding M, Sampson L, Willett WC, Manson JE, Wang M, Rosner B, Hu FB, Sun Q (2020). Intake of whole grain foods and risk of type 2 diabetes: results from three prospective cohort studies. BMJ.

[5] Herforth A, Arimond M, Álvarez-Sánchez C, Coates J, Christianson K, Muehlhoff E (2019). A global review of food-based dietary guidelines. Adv Nutr.

[6] Ordunez P, Mize V, Barbosa M, Legetic B, Hennis AJ (2015). A rapid assessment study on the implementation of a core set of interventions to improve cardiovascular health in Latin America and the Caribbean. Glob Heart.

[7] Sisa I, Abeyá-Gilardon E, Fisberg RM, Jackson MD, Mangialavori GL, Sichieri R, Cudhea F, Bannuru RR, Ruthazer R, Mozaffarian D, Singh GM (2020). Impact of diet on CVD and diabetes mortality in Latin America and the Caribbean: a comparative risk assessment analysis. Public Health Nutr.

[8] Suthers R, Broom M, Beck, (2018). Key characteristics of public health interventions aimed at increasing whole grain intake: a systematic review. J Nutr Educ Behav.

[9] Fisberg M, Kovalskys I, Gómez G, Rigotti A, Cortés LY, Herrera-Cuenca M, YépezGarcía MC, Pareja RG, Guajardo V, Zimberg IZ, NogueiraPrevidelli A, Koletzko B, ELANS Study Group (2018). Energy intake and food sources of eight Latin American countries: results from the Latin American Study of Nutrition and Health (ELANS). Public Health Nutr.

[10] Martorell R, de Romaña DL (2017). Components of successful staple food fortification programs: lessons from Latin America. Food Nutr Bull.

[11] Popkin BM, Reardon T (2018). Obesity and the food system transformation in Latin America. Obes Rev.

[12] Fisberg M, Kovalskys I, Gómez G, Rigotti A, Cortés LY, Herrera-Cuenca M, Yépez MC, Pareja RG, Guajardo V, Zimberg IZ, ChiavegattoFilho ADP, Pratt M, Koletzko B, Tucker KL, ELANS Study Group (2016). Latin American Study of Nutrition and Health (ELANS): rationale and study design. BMC Public Health.

[13] Kovalskys I, Fisberg M, Gómez G, RigottiCortés ALY, Yépez MC, Pareja RG, Herrera-Cuenca M, Zimberg IZ, Tucker KL, Koletzko B, Pratt M, ELANS Study Group (2015). Standardization of the food composition database used in the Latin American Nutrition and Health Study (ELANS). Nutrients.

[14] Moshfegh AJ, Rhodes DG, Baer DJ, Murayi T, Clemens JC, Rumpler WV, Paul DR, Sebastian RS, Kuczynski KJ, Ingwersen LA, Staples RC, Cleveland LE (2008). The US Department of Agriculture Automated Multiple-Pass Method reduces bias in the collection of energy intakes. Am J Clin Nutr.

[15] U.S. Department of Agriculture, Agricultural Research Service (2013) USDA National Nutrient Database for Standard Reference, Release 26. Nutrient Data Laboratory Home Page, https://www.ars.usda.gov/ba/bhnrc /ndl. Accessed on 30 Mar 2020

[16] Ross AB, Kristensen M, Seal CJ, Jacques P, McKeown NM (2015). Recommendations for reporting whole-grain intake in observational and intervention studies. Am J Clin Nutr.

[17] Tooze JA, Kipnis V, Buckman DW, Carroll RJ, Freedman LS, Guenther PM, Krebs-Smith SM, Subar AF, Dodd KW (2010). A mixed-effects model approach for estimating the distribution of usual intake of nutrients: the NCI method. Stat Med.

[18] Willett W (2013). Nutritional epidemiology.

[19] Ross AB, van der Kamp JW, King R, Lê KA, Mejborn H, Seal CJ, Thielecke F, Forum H (2017). Perspective: a definition for whole-grain food products-recommendations from the healthgrain forum. Adv Nutr.

[20] Oliveira MSDS, Arceño MA, Sato PM, Scagliusi FB (2019). Comparison of government recommendations for healthy eating habits in visual representations of food-based dietary guidelines in Latin America. Cad Saude Publica.

[21] Ministerio de Salud (2014) Guías de alimentación sana. Ministerio de Salud Santiago

[22] Bonvecchio Arenas A, Fernández-Gaxiola AC, Belausteguigoitia MP, Kaufer-Horwitz M, Pérez Lizaur AB, Rivera Dommarco JAR, editores (2015) Guías alimentarias y de actividad física en contexto de sobrepeso y obesidad en la población Mexicana. Intersistemas, Ciudad de México

[23] Ministerio de Salud Pública y Bienestar Social (2015) La olla nutricional paraguaya: guías alimentarias del Paraguay. Instituto Nacional de Alimentación y Nutrición, Asunción

[24] Ministerio de Salud Pública; Organización Panamericana de la Salud (2016) Guía alimentaria para la población uruguaya: para una alimentación saludable, compartida y placentera. Ministerio de Salud Pública, Montevideo

[25] Dirección General de Promoción de la Salud, Ministerio de Salud (2013) Bases técnicas de las guías alimentarias para la población boliviana. Ministerio de Salud/Editorial Quatro Hnos, La Paz

[26] Buyken AE, Mela DJ, Dussort P, Johnson IT, Macdonald IA, Stowell JD, Brouns F (2018). Dietary carbohydrates: a review of international recommendations and the methods used to derive them. Eur J Clin Nutr.

[27] Mathews R, Chu Y (2020). Global review of whole grain definitions and health claims. Nutr Rev.

[28] Rehm CD, Penalvo JL, Afshin A, Mozaffarian D (2016). Dietary intake among US Adults, 1999–2012. JAMA.

[29] Kyrø C, Skeie G, Dragsted LO, Christensen J, Overvad K, Hallmans G, Johansson I, Lund E, Slimani N, Johnsen NF, Halkjær J, Tjønneland A, Olsen A (2012). Intake of whole grain in Scandinavia: intake, sources and compliance with new national recommendations. Scand J Public Health.

[30] Bellisle F, Hébel P, Colin J, Reyé B, Hopkins S (2014). Consumption of whole grains in French children, adolescents and adults. Br J Nutr.

[31] Kissock KR, Neale EP, Beck EJ (2020). The relevance of whole grain food definitions in estimation of whole grain intake: a secondary analysis of the National Nutrition and Physical Activity Survey 2011–2012. Public Health Nutr.

[32] Seal CJ, Nugent AP, Tee ES, Thielecke F (2016). Whole-grain dietary recommendations: the need for a unified global approach. Br J Nutr.

[33] Mello AV, Sarti FM, Pereira JL, Goldbaum M, Cesar CLG, Alves MCGP, Fisberg RM (2018). Determinants of inequalities in the quality of Brazilian diet: trends in 12 year population-based study (2003–2015). Int J Equity Health.

[34] Tester JM, Leung CW, Leak TM, Laraia BA (2017). Recent uptrend in whole-grain intake is absent for low-income adolescents, National Health and Nutrition Examination Survey, 2005–2012. Prev Chronic Dis.

[35] Mann KD, Pearce MS, McKevith B, Thielecke F, Seal CJ (2015). Low whole grain intake in the UK: results from the National Diet and Nutrition Survey rolling programme 2008–11. Br J Nutr.

[36] Harriman C (2012) Shrinking the price gap for whole grains. Paper presented at: Whole Grain Summit, AACC International. Available at: https://www.aaccnet.org/publications/plexus/cfwplexus/library/books/Documents/WholeGrainsSummit2012/CPLEX-2013-1001-17B.pdf. Accessed on Sept 14, 2020

[37] Mora-García G, Ruiz-Díaz MS, Villegas R, García-Larsen V (2020). Changes in diet quality over 10 years of nutrition transition in Colombia: analysis of the 2005 and 2015 nationally representative cross-sectional surveys. Int J Public Health.

[38] Shan Z, Rehm CD, Rogers G, Ruan M, Wang DD, Hu FB, Mozaffarian D, Zhang FF, Bhupathiraju SN (2019). Trends in dietary carbohydrate, protein, and fat intake and diet quality among US adults, 1999–2016. JAMA.

[39] Liu J, Rehm CD, Onopa J, Mozaffarian D (2020). Trends in diet quality among youth in the United States, 1999–2016. JAMA.

[40] Imamura F, Micha R, Khatibzadeh S, Fahimi S, Shi P, Powles J, Mozaffarian D (2015). Dietary quality among men and women in 187 countries in 1990 and 2010: a systematic assessment. Lancet Glob Health.

[41] Christoph MJ, Larson NI, Winkler MR, Wall MM, Neumark-Sztainer D (2019). Longitudinal trajectories and prevalence of meeting dietary guidelines during the transition from adolescence to young adulthood. Am J Clin Nutr.

[42] Toups KE (2020). Global approaches to promoting whole grain consumption. Nutr Rev.

[43] FAO – Food and Agriculture Organization (2017) América Latina e no caribe: Panorama da segurança alimentar e nutricional. Sistemas alimentares sustentáveis para acabar com a fome e a má nutrição. 2017. [Latin America and the Caribbean: Overview of food and nutritional security. Sustainable food systems to end hunger and malnutrition]. http://www.fao.org/3/a-i6977o.pdf. Accessed on 18 de Sept 2020.

[44] FAO - Food and Agriculture Organization (2018) Estudio para identificar y analizar experiencias nacionales relacionadas que fomenten el bienestar nutricional en América Latina y el Caribe. [Study to identify and analyze national related experiences to foster nutritional wellness in Latin America and the Caribbean]. http://www.fao.org/3/i8901es/I8901ES.pdf . Accessed on 18 Sept 2020

[45] USDA - United States Department of Agriculture Food and Nutrition Service (2013) SP 02 -2013 Corn Masa (Dough) for Use in Tortilla Chips, Taco Shells, and Tamales. https://childnutrition.ncpublicschools.gov/regulations-policies/usda-policy-memos/2013/sp-02-2013.pdf. Accessed on 8 Jun 2021

[46] Oldways Whole Grain Council. Corn – October Grain of the Month https://wholegrainscouncil.org/whole-grains-101/grain-month-calendar/corn-%E2%80%93-october-grain-month. Accessed on 8 Jun 2021

[47] Galea LM, Dalton SMC, Beck EJ, Cashman CJ, Probst YC (2016). Update of a database for estimation of whole grain content of foods in Australia. J Food Compost Anal.

[48] Brazil. Proposal for Public Consultation. Process n: 25351.715085/2015–78. Proposed Resolution of the Collegiate Board that provides for the requirements for identification as whole grain and for highlighting the whole grain ingredients in the labeling of foods containing cereals. Brasília, Federal District: Union Official Journal; April 8^th^ 2020, p 124. Accessed on 18 Sept 2020 [In Portuguese]

[49] Prates SMS, Sabion NA, Nespolo JS, Alves L, Anastácio LR (2020). Labeling and classification of breads, biscuits and toasts sold as wholegrain in Brazil. Res Soc Dev.

[50] Liu J, Rehm CD, Shi P, McKeown NM, Mozaffarian D, Micha R (2020). A comparison of different practical indices for assessing carbohydrate quality among carbohydrate-rich processed products in the US. PLoS ONE.

[51] Tan D, Olden AN, Orengo A, Francey C, Campos VC, Fayet-Moore F, Kim JE, Lê KA (2020). An assessment of three carbohydrate metrics of nutritional quality for packaged foods and beverages in Australia and Southeast Asia. Nutrients.

[52] Lanuza F, Zamora-Ros R, Hidalgo-Liberona N, Andrés-Lacueva C, Meroño T (2020). Wholegrain consumption and risk factors for cardiorenal metabolic diseases in Chile: a cross-sectional analysis of 2016–2017 Health National Survey. Nutrients.

[53] Fisberg M, Kovalskys I, Gómez G, Rigotti A, Cortés LY, Herrera-Cuenca M, Yépez MC, Pareja RG, Guajardo V, Zimberg IZ, DelArco A, Zonis L, Previdelli AN, Guajardo V, Moreno LA, Fisberg R, ELANS Study Group (2019). Latin American consumption of major food groups: Results from the ELANS study. PLoS ONE.

[54] Department of Agriculture, Philippine Rice Research Institute. Be Ricesponsible. http://www.philrice.gov.ph/campaign/be-riceponsible/. Accessed on 26 Sept 2020

