# Correction to: Total and whole grain intake in Latin America: fndings from the multicenter cross-sectional Latin American Study of Health and Nutrition (ELANS)

**DOI:** 10.1007/s00394-021-02723-9

**Published:** 2021-11-08

**Authors:** Regina Mara Fisberg, Mariane Mello Fontanelli, Irina Kowalskys, Georgina Gómez, Attilio Rigotti, Lilia Yadira Cortés, Martha Yépez García, Rossina G. Pareja, Marianella Herrera-Cuenca, Mauro Fisberg, ELANS Study Group, Mauro Fisberg, Irina Kovalskys, Georgina Gómez Salas, Attilio Rigotti, Lilia Yadira Cortés Sanabria, Martha Cecilia Yépez García, Rossina Gabriella Pareja Torres, Marianella Herrera-Cuenca, Berthold Koletzko, Luis A. Moreno, Michael Pratt, Regina Mara Fisberg, Agatha Nogueira Previdelli, Viviana Guajardo, Ioná Zalcman Zimberg, Viviana Guajardo, María Paz Amigo, Ximena Janezic, Fernando Cardini, Myriam Echeverry, Natasha Aparecida Grande de França, Guadalupe Echeverría, Leslie Landaeta, Óscar Castillo, Luz Nayibe Vargas, Luisa Fernanda Tobar Yuri Milena Castillo, Rafael Monge Rojas, Mónica Villar Cáceres, María Belén Ocampo, María Reyna Liria, Krysty Meza, Mellisa Abad, Marianella Herrera- Cuenca, Maritza Landaeta-Jiménez, Betty Méndez, Maura Vásquez, Guillermo Ramírez, Pablo Hernández, Carmen Meza, Omaira Rivas, Vanessa Morales, Priscila Bezerra Gonçalves, Claudia Alberico, Gerson Luis de Moraes Ferrari

**Affiliations:** 1 Department of Nutrition, School of Public Health, University of São Paulo, Sao Paulo, 01246-904 Brazil; 2 Facultad de Ciencias Médicas, Carrera de Nutrición, Pontificia Universidad Católica Argentina, C1107 AAZ Buenos Aires, Argentina; 3 Departamento de Bioquímica, Escuela de Medicina, Universidad de Costa Rica, 11501-2060 San Jose, Costa Rica; 4 Centro de Nutrición Molecular y Enfermedades Crónicas, Departamento de Nutrición, Diabetes y Metabolismo, Escuela de Medicina, Pontificia Universidad Católica, 8330024 Santiago, Chile; 5 Departamento de Nutrición y Bioquímica, Pontificia Universidad Javeriana, 110231 Bogota, Colombia; 6 Colégio de Ciencias de la Salud, Universidad San Francisco de Quito, Quito, 17-1200-841 Ecuador; 7 Instituto de Investigación Nutricional, Lima, 15026 Peru; 8 Centro de Estudios del Desarrollo, Universidad Central de Venezuela (CENDES-UCV)/Fundación Bengoa, Caracas, 1053 Venezuela; 9 Fundação José Luiz Egydio Setubal, Instituto Pensi, Hospital Infantil Sabará, Sao Paulo, 01227-200 Brazil

## Correction to: European Journal of Nutrition 10.1007/s00394-021-02635-8

The article Total and whole grain intake in Latin America: fndings from the multicenter cross-sectional Latin American Study of Health and Nutrition (ELANS), written by Regina Mara Fisberg, Mariane Mello Fontanelli, Irina Kowalskys, Georgina Gómez, Attilio Rigotti, Lilia Yadira Cortés, Martha Yépez García, Rossina G. Pareja, Marianella Herrera-Cuenca, Mauro Fisberg and ELANS Study Group, was originally published electronically on the publisher’s internet portal on 20 April 2020 without open access. With the author(s)’ decision to opt for Open Choice the copyright of the article changed on 12 October 2021 to © The Author(s) 2021 and this article is licensed under a Creative Commons Attribution 4.0 International License, which permits use, sharing, adaptation, distribution and reproduction in any medium or format, as long as you give appropriate credit to the original author(s) and the source, provide a link to the Creative Commons licence, and indicate if changes were made. The images or other third party material in this article are included in the article's Creative Commons licence, unless indicated otherwise in a credit line to the material. If material is not included in the article's Creative Commons licence and your intended use is not permitted by statutory regulation or exceeds the permitted use, you will need to obtain permission directly from the copyright holder. To view a copy of this licence, visit http://creativecommons.org/licenses/by/4.0/.

The original article has been corrected.

