# Supplementary material for: Total and whole grain intake in Latin America: findings from the multicenter cross-sectional Latin American Study of Health and Nutrition (ELANS)
Source: Eur J Nutr. 2021 Jul 7;61(1):489–501. doi: 10.1007/s00394-021-02635-8 (PMC8783851; doi:10.1007/s00394-021-02635-8)
Supplement: Supplementary file 1 — Supplementary file1 (DOCX 52 KB) [file 394_2021_2635_MOESM1_ESM.docx]

**European Journal of Nutrition**

**Online Resource**

**Total and whole grain intake in Latin America: findings from the multicenter cross-sectional Latin American Study of Health and Nutrition (ELANS)**

Regina Mara Fisberg, Mariane Mello Fontanelli, Irina Kowalskys, Georgina Gómez, Attilio Rigotti, Lilia Yadira Cortés, Martha Yépez García, Rossina G. Pareja, Marianella Herrera-Cuenca, Mauro Fisberg on behalf of the ELANS Study Group

Sumário

[Classification of grain foods 2](#_Toc74146422)

[**Online resource Table 1.** List of grain ingredients of Nutrition Data System for Research (NDSR) software 2](#_Toc74146423)

[**Online Resource Table 2.** Total grain foods, grain foods containing whole grains and grain foods containing >50% whole grains in the Latin American Study of Nutrition and Health (ELANS) database according to country, 2015. 4](#_Toc74146424)

[**Online Resource Table 3.** Energy adjusted dietary intake (g/2000 kcal/d) of grain food groups according to sociodemographic characteristics and country based on the Latin American Study of Nutrition and Health (ELANS), 2015. 5](#_Toc74146425)

[**Online Resource Table 4.** Proportion grain foods containing whole grains to total grain foods and the proportion of foods containing >50% whole grains to total grain foods consumed in Latin American countries based on the Latin American Study of Nutrition and Health (ELANS), 2015. 8](#_Toc74146426)

[**Online Resource Table 5.** Coefficients and 95% confidence intervals for the association of sociodemographic variables and the intake of total grain foods, foods containing whole grains, and foods containing >50% whole grains in Latin American countries based on the Latin American Study of Nutrition and Health (ELANS), 2015. 9](#_Toc74146427)

[References 12](#_Toc74146428)

# Classification of grain foods

The definition adopted in this study is in line with the global proposed definitions of whole-grain ingredient and whole-grain food [1,2]. First, the USDA “grain products” food group was used to identify grain foods. Then, grain products were categorized in the following groups: (1) total grain foods, (2) grain foods containing whole grains (WG, any amount), and (3) grain foods containing >50% WG. Total grains (refined grains plus WG) included all food items classified as “grain products”. For grain foods containing WG and grain foods containing >50% WG, the “total grain (ounce equivalent)” and “whole grain (ounce equivalent)” NDSR variables were used to estimate a whole-grain to total-grain proportion. Foods with the proportion between 0 and 50% were categorized as foods containing any amount of whole grains, and foods with the whole-grain to total-grain proportion more than 50%, were categorized as foods containing >50% WG. A detailed list of refined and whole grain ingredients is presented below.

Online Resource Table 1. List of grain ingredients of Nutrition Data System for Research (NDSR) software. Source: Nutrition Coordinating Center (NCC), University of Minnesota, Minneapolis, MN (2014) Nutrition Data System for Research online manual. <https://drive.google.com/file/d/0B4snm2Q3-ffQbkdHejJ2MWZNWjQ/view> Accessed 08 June 2021

| **Grain** | **Whole grain ingredients** | **Refined grain ingredients** |
| --- | --- | --- |
| Amaranth | Amaranth |  |
| Barley | Barley malt | Barley |
|  | Dehulled barley | Barley bits |
|  | Flaked barley | Barley flakes |
|  | Hulled barley | Barley flour |
|  | Malt barley | Barley germ |
|  | Malted barley flour | Barley grits |
|  | Sprouted barley flour | Milled barley |
|  | Whole barley | Pearled barley |
|  | Whole grain barley flour |  |
| Buckwheat | Buckwheat groats | Buckwheat flour (refined) |
|  | Buckwheat flour (whole groats) |  |
| Corn | Corn | Corn bran |
|  | Masa harina | Corn flour |
|  | Whole corn | Corn grits |
|  | Whole corn flour | Corn masa |
|  | Whole grain corn meal | Corn meal |
|  |  | Degermed cornmeal |
| Millet | Millet |  |
| Oats | Cracked oats | Oat bran |
|  | Oat flour | Oat fiber |
|  | Oatmeal |  |
|  | Oats |  |
|  | Rolled oats |  |
|  | Steel cut oats |  |
|  | Whole oat flour |  |
| Popcorn | Popcorn |  |
| Rice | Brown rice | Rice bran |
|  | Brown rice flour | Rice flour |
|  | Brown rice meal | White rice |
| Rye | Dark rye flour | Cracked rye |
|  | Rolled rye | Dark rye meal |
|  | Rye berries | Ground rye |
|  | Rye flakes | Rye |
|  | Rye kernels | Rye flour |
|  | Whole grain rye flour | Rye meal |
|  | Whole rolled rye meal | White rye flour |
|  | Whole rye meal |  |
|  | Whole rye flour |  |
| Sorghum | Whole sorghum |  |
| Spelt | Spelt |  |
| Teff | Teff |  |
| Triticale | Whole grain triticale | Triticale flour |
| Quinoa | Quinoa |  |
| Wheat | Bulgur | Wheat bran |
|  | Cracked wheat | Wheat fiber |
|  | Crushed wheat | Wheat flour – all types (bread, cake, all– purpose, enriched, unenriched, unbleached, high gluten) |
|  | Flaked wheat | Wheat germ |
|  | Graham flour | Wheat gluten |
|  | Rolled wheat | Wheat nuggets |
|  | Rolled whole wheat | Whole wheat bran |
|  | Stone ground whole wheat flour |  |
|  | Wheat berries |  |
|  | Wheat kernels |  |
|  | Whole wheat flour |  |
| Wild rice | Wild rice |  |
|  | Wild rice flour |  |

# Online Resource Table 2. Total grain foods, grain foods containing whole grains and grain foods containing >50% whole grains in the Latin American Study of Nutrition and Health (ELANS) database according to country, 2015.

| Countries | Grain foods | | Grain foods containing WG | | Grain foods containing >50% WG | |
| --- | --- | --- | --- | --- | --- | --- |
|  | n | % | n | % | n | % |
| ELANS | 695 | 100.0 | 206 | 29.6 | 148 | 21.3 |
| Argentina | 217 | 100.0 | 56 | 25.8 | 41 | 18.9 |
| Brazil | 185 | 100.0 | 48 | 25.9 | 35 | 18.9 |
| Chile | 172 | 100.0 | 58 | 33.7 | 43 | 25.0 |
| Colombia | 213 | 100.0 | 82 | 38.5 | 64 | 30.0 |
| Costa Rica | 193 | 100.0 | 71 | 36.8 | 55 | 28.5 |
| Ecuador | 145 | 100.0 | 51 | 35.2 | 41 | 28.3 |
| Peru | 156 | 100.0 | 41 | 26.3 | 29 | 18.6 |
| Venezuela | 126 | 100.0 | 30 | 23.8 | 21 | 16.7 |

WG whole grains, n number of food products

# Online Resource Table 3. Energy adjusted dietary intake (g/2000 kcal/d) of grain food groups according to sociodemographic characteristics and country based on the Latin American Study of Nutrition and Health (ELANS), 2015.

| **Sociodemographic** | **Total grains** | | **Foods containing whole grains** | | **Foods ≥50% whole grains** | |
| --- | --- | --- | --- | --- | --- | --- |
|  | **Mean (SD)** | **Median (IQR)** | **Mean (SD)** | **Median (IQR)** | **Mean (SD)** | **Median (IQR)** |
|  | **ELANS (n=9218)** | |  |  |  |  |
| Overall (g/2000kcal/d) | 321.5 (90.8) | 302.3 (107.4) | 14.9 (12.4) | 10.8 (12.5) | 12.5 (10.7) | 8.8 (10.6) |
| Overall adjusted ^a^ | 318.6 (9.6) | 319.4 (16.8) | 14.7 (2.6) | 15.0 (4.0) | 12.2 (1.8) | 12.2 (2.6) |
| Age group, years | | | | | | |
| 15-19 | 334.6 (93.7) | 313.9 (109.7) | 13.8 (11.1) | 9.5 (11.5) | 11.6 (9.6) | 8.3 (9.9) |
| 20-34 | 323.5 (91.3) | 302.9 (106.3) | 13.9 (11.6) | 10.0 (11.2) | 11.7 (9.7) | 8.4 (9.3) |
| 35-49 | 319.4 (91.0) | 300.7 (110.0) | 14.7 (11.9) | 10.9 (12.4) | 12.4 (10.3) | 8.7 (10.5) |
| 50-65 | 312.2 (86.7) | 297.1 (105.3) | 17.4 (14.8) | 13.1 (16.1) | 14.7 (13.0) | 10.8 (13.3) |
| Sex |  |  |  |  |  |  |
| Male | 326.8 (94.9 | 306.3 (110.9) | 12.7 (11.1) | 9.1 (10.8) | 11.1 (9.9) | 7.7 (9.6) |
| Female | 316.6 (86.7) | 298.8 (104.7) | 16.8 (13.3) | 12.5 (14.2) | 13.7 (11.2) | 10.1 (11.7) |
| Socioeconomic level | | | | | | |
| High | 339.6 (99.5) | 319.5 (138.6) | 17.3 (15.4) | 12.3 (14.2) | 14.5 (13.5) | 9.9 (12.4) |
| Medium | 318.4 (86.9) | 301.8 (107.3) | 15.0 (13.1) | 10.9 (13.2) | 12.3 (11.1) | 8.5 (10.6) |
| Low | 320.4 (91.7) | 300.8 (103.2) | 14.3 (11.3) | 10.4 (11.6) | 12.3 (9.7) | 8.9 (10.3) |
|  | **Argentina (n=1266)** | |  |  |  |  |
| Overall (g/d) | 253.2 (46.6) | 249.3 (62.2) | 14.4 (10.8) | 10.1 (10.8) | 11.1 (10.0) | 7.5 (6.9) |
| Overall adjusted | 254.8 (12.6) | 253.9 (16.9) | 14.4 (4.4) | 14.3 (6.4) | 11.1 (3.6) | 10.8 (5.4) |
| Age group, years |  |  |  |  |  |  |
| 15-19 | 275.9 (47.1) | 272.0 (66.9) | 8.7 (6.3) | 6.0 (3.2) | 6.4 (5.6) | 4.4 (2.1) |
| 20-34 | 260.8 (45.0) | 257.4 (59.7) | 11.9 (8.9) | 8.0 (8.6) | 9.0 (7.9) | 6.1 (3.6) |
| 35-49 | 246.5 (44.1) | 240.3 (58.9) | 14.7 (9.6) | 10.5 (10.6) | 11.8 (9.3) | 8.0 (6.5) |
| 50-65 | 238.3 (45.3) | 234.2 (57.0) | 20.6 (13.5) | 14.8 (16.4) | 16.0 (11.9) | 11.3 (10.6) |
| Sex |  |  |  |  |  |  |
| Male | 250.4 (45.1) | 246.4 (62.5) | 11.8 (9.2) | 8.1 (7.6) | 9.0 (8.3) | 6.0 (4.6) |
| Female | 255.5 (47.7) | 250.8 (62.5) | 16.5 (11.5) | 12.2 (12.3) | 12.9 (10.3) | 8.8 (8.5) |
| Socioeconomic level | | | | | | |
| High | 248.1 (41.7) | 247.2 (55.6) | 17.4 (13.8) | 12.2 (14.6) | 14.3 (13.4) | 8.8 (12.3) |
| Medium | 249.9 (46.9) | 243.8 (62.7) | 15.1 (11.1) | 10.9 (12.2) | 11.6 (10.1) | 7.9 (7.3) |
| Low | 256.9 (46.6) | 254.7 (62.8) | 13.3 (10.0) | 9.3 (8.9) | 10.4 (8.6) | 7.1 (6.4) |
|  | **Brazil (n=2000)** | |  |  |  |  |
| Overall (g/d) | 299.2 (62.1) | 298.3 (79.0) | 10.4 (14.6) | 5.6 (2.9) | 8.2 (11.6) | 4.7 (1.9) |
| Overall adjusted | 298.5 (9.9) | 301.5 (10.6) | 10.6 (2.3) | 10.8 (3.8) | 8.2 (1.6) | 8.2 (2.2) |
| Age group, years |  |  |  |  |  |  |
| 15-19 | 303.7 (57.5) | 308.4 (79.0) | 10.9 (13.7) | 5.8 (3.4) | 8.5 (10.8) | 4.8 (2.2) |
| 20-34 | 300.2 (62.9) | 299.5 (77.8) | 10.2 (14.6) | 5.6 (2.9) | 7.9 (11.1) | 4.7 (1.9) |
| 35-49 | 299.9 (62.2) | 299.3 (80.8) | 9.9 (13.2) | 5.6 (2.8) | 7.7 (10.1) | 4.7 (1.8) |
| 50-65 | 293.6 (62.9) | 294.0 (77.3) | 11.3 (16.9) | 5.6 (2.6) | 9.3 (14.6) | 4.8 (1.7) |
| Sex |  |  |  |  |  |  |
| Male | 304.3 (63.6) | 302.3 (80.5) | 8.5 (13.3) | 4.7 (1.7) | 7.0 (11.0) | 4.2 (1.3) |
| Female | 294.6 (60.4) | 293.6 (78.3) | 12.1 (15.5) | 6.4 (3.6) | 9.2 (12.1) | 5.2 (2.0) |
| Socioeconomic level |  |  |  |  |  |  |
| High | 280.2 (56.1) | 282.4 (70.3) | 13.9 (21.0) | 5.6 (7.0) | 11.4 (18.1) | 4.6 (2.4) |
| Medium | 296.9 (60.0) | 295.5 (78.2) | 10.5 (14.6) | 5.6 (2.8) | 8.0 (11.4) | 4.7 (1.8) |
| Low | 304.9 (64.4) | 305.1 (80.0) | 9.6 (13.0) | 5.6 (2.8) | 7.7 (10.2) | 4.8 (1.9) |
|  | **Chile (n=879)** | |  |  |  |  |
| Overall (g/d) | 304.4 (49.2) | 302.4 (64.3) | 12.1 (15.8) | 5.9 (4.5) | 9.0 (12.8) | 5.0 (2.5) |
| Overall adjusted | 301.4 (7.2) | 301.5 (12.3) | 11.8 (3.8) | 11.3 (5.2) | 8.9 (2.3) | 8.6 (3.4) |
| Age group, years |  |  |  |  |  |  |
| 15-19 | 310.1 (50.6) | 304.7 (66.6) | 12.9 (15.1) | 6.4 (9.0) | 9.7 (12.2) | 5.3 (3.3) |
| 20-34 | 297.8 (48.9) | 297.7 (65.7) | 12.4 (16.1) | 6.1 (4.4) | 8.9 (11.7) | 5.0 (2.6) |
| 35-49 | 305.0 (50.3) | 301.4 (64.2) | 11.6 (16.0) | 5.8 (4.1) | 8.8 (13.4) | 4.9 (2.4) |
| 50-65 | 310.4 (46.4) | 313.1 (63.1) | 11.7 (15.7) | 5.5 (3.5) | 9.1 (13.8) | 4.8 (2.3) |
| Sex |  |  |  |  |  |  |
| Male | 300.4 (47.9) | 299.9 (62.1) | 9.4 (13.6) | 4.5 (1.6) | 7.4 (11.6) | 4.1 (1.3) |
| Female | 308.1 (50.1) | 303.4 (66.3) | 14.5 (17.3) | 7.0 (8.9) | 10.5 (13.6) | 5.8 (2.4) |
| Socioeconomic level |  |  |  |  |  |  |
| High | 305.4 (50.1) | 306.1 (66.1) | 18.3 (23.4) | 7.0 (16.0) | 13.1 (18.7) | 5.4 (5.7) |
| Medium | 301.4 (48.2) | 298.1 (65.5) | 13.4 (16.7) | 6.2 (11.0) | 9.8 (13.7) | 5.2 (3.2) |
| Low | 307.1 (49.8) | 304.9 (62.5) | 9.5 (12.4) | 5.5 (2.9) | 7.5 (9.8) | 4.7 (2.2) |
|  | **Colombia (n=1230)** | |  |  |  |  |
| Overall (g/d) | 276.1 (47.2) | 275.2 (62.4) | 19.4 (11.1) | 16.0 (12.1) | 17.2 (8.9) | 14.4 (9.8) |
| Overall adjusted | 275.5 (7.6) | 275.0 (8.7) | 19.6 (3.6) | 19.6 (5.0) | 17.4 (2.5) | 17.0 (3.0) |
| Age group, years |  |  |  |  |  |  |
| 15-19 | 272.3 (43.5) | 278.3 (60.4) | 15.5 (9.2) | 12.1 (10.1) | 14.0 (6.9) | 11.4 (8.4) |
| 20-34 | 274.9 (44.6) | 273.6 (61.7) | 17.7 (9.9) | 14.4 (10.0) | 15.9 (8.2) | 13.1 (8.6) |
| 35-49 | 275.3 (47.5) | 275.0 (61.7) | 20.1 (10.2) | 17.4 (12.6) | 17.9 (8.1) | 15.1 (10.7) |
| 50-65 | 280.5 (51.8) | 276.5 (70.4) | 23.1 (13.2) | 18.2 (14.4) | 20.0 (10.6) | 16.6 (11.3) |
| Sex |  |  |  |  |  |  |
| Male | 279.6 (47.0) | 280.8 (62.7) | 16.8 (10.0) | 14.0 (9.6) | 15.7 (8.6) | 13.2 (8.1) |
| Female | 272.6 (47.1) | 268.8 (62.0) | 21.9 (11.6) | 18.0 (14.7) | 18.7 (8.9) | 15.8 (11.4) |
| Socioeconomic level |  |  |  |  |  |  |
| High | 260.4 (41.2) | 258.9 (49.8) | 20.1 (10.3) | 16.6 (15.6) | 17.6 (8.1) | 15.2 (13.3) |
| Medium | 272.6 (44.9) | 272.3 (59.0) | 20.6 (12.7) | 16.5 (12.8) | 18.2 (10.2) | 14.8 (10.7) |
| Low | 279.1 (48.4) | 279.0 (65.0) | 18.8 (10.3) | 15.5 (11.5) | 16.7 (8.2) | 14.1 (8.9) |
|  | **Costa Rica (n=798)** | |  |  |  |  |
| Overall (g/d) | 389.1 (71.4) | 387.9 (95.1) | 20.8 (9.8) | 17.9 (11.3) | 18.9 (9.2) | 15.7 (10.7) |
| Overall adjusted | 389.1 (25.2) | 384.1 (32.2) | 20.8 (2.8) | 21.4 (4.0) | 18.9 (2.1) | 18.5 (2.4) |
| Age group, years |  |  |  |  |  |  |
| 15-19 | 380.5 (73.3) | 376.7 (88.4) | 18.7 (7.6) | 16.3 (10.2) | 17.0 (7.3) | 14.4 (9.8) |
| 20-34 | 370.9 (69.2) | 367.4 (96.6) | 19.7 (9.5) | 16.5 (11.0) | 17.8 (8.9) | 14.3 (10.4) |
| 35-49 | 403.9 (69.9) | 405.6 (95.9) | 21.1 (10.4) | 18.0 (11.0) | 19.1 (9.7) | 15.8 (10.3) |
| 50-65 | 410.2 (66.7) | 404.7 (94.0) | 24.4 (10.2) | 22.3 (14.5) | 22.0 (9.8) | 19.6 (11.8) |
| Sex |  |  |  |  |  |  |
| Male | 397.9 (72.9) | 395.3 (97.8) | 18.6 (8.5) | 15.5 (10.5) | 17.6 (8.4) | 14.5 (10.1) |
| Female | 380.5 (69.0) | 377.1 (93.6) | 23.0 (10.6) | 19.9 (12.4) | 20.1 (9.8) | 16.8 (11.4) |
| Socioeconomic level |  |  |  |  |  |  |
| High | 367.6 (64.4) | 365.3 (97.0) | 22.8 (10.1) | 20.6 (13.7) | 21.0 (9.6) | 18.5 (13.4) |
| Medium | 381.9 (70.5) | 377.9 (90.3) | 20.4 (10.1) | 17.2 (10.8) | 18.4 (9.3) | 15.2 (10.1) |
| Low | 409.7 (71.1) | 411.1 (102.4) | 20.7 (9.1) | 18.1 (10.7) | 18.8 (8.8) | 15.9 (10.0) |
|  | **Ecuador (n=800)** | |  |  |  |  |
| Overall (g/d) | 347.9 (56.9) | 346.9 (75.2) | 14.4 (9.9) | 11.4 (8.4) | 12.4 (8.9)6 | 9.6 (7.6) |
| Overall adjusted | 347.9 (16.2) | 349.9 (19.8) | 14.3 (5.0) | 14.0 (8.3) | 12.4 (4.6) | 12.1 (7.5) |
| Age group, years |  |  |  |  |  |  |
| 15-19 | 365.3 (52.0) | 362.5 (70.6) | 10.2 (5.7) | 8.4 (4.8) | 8.4 (4.9) | 6.8 (4.0) |
| 20-34 | 349.4 (57.7) | 347.8 (68.9) | 11.9 (6.8) | 9.9 (6.1) | 10.1 (6.1) | 8.2 (5.8) |
| 35-49 | 343.4 (57.8) | 346.2 (82.6) | 15.7 (9.5) | 13.0 (8.1) | 13.5 (7.9) | 10.9 (7.2) |
| 50-65 | 335.5 (54.2) | 331.7 (73.5) | 22.3 (14.0) | 17.4 (13.7) | 20.1 (13.3) | 15.3 (13.3) |
| Sex |  |  |  |  |  |  |
| Male | 356.3 (56.8) | 355.5 (74.4) | 11.2 (7.6) | 8.6 (5.8) | 9.6 (6.5) | 7.4 (5.2) |
| Female | 339.7 (55.9) | 341.8 (71.6) | 17.6 (10.8) | 14.1 (9.4) | 15.2 (10.1) | 12.0 (8.7) |
| Socioeconomic level |  |  |  |  |  |  |
| High | 321.6 (56.7) | 321.9 (71.4) | 16.3 (11.6) | 12.4 (11.2) | 14.7 (11.0) | 10.8 (10.6) |
| Medium | 346.3 (56.0) | 346.7 (74.7) | 14.7 (10.2) | 11.6 (8.3) | 12.5 (8.8) | 10.1 (6.7) |
| Low | 356.0 (55.6) | 353.6 (69.5) | 13.7 (9.0) | 11.0 (8.0) | 11.8 (8.3) | 9.3 (7.5) |
|  | **Peru (n=1113)** | |  |  |  |  |
| Overall (g/d) | 483.9 (72.2) | 485.4 (96.2) | 17.0 (10.6) | 13.5 (12.7) | 13.7 (9.5) | 10.3 (11.0) |
| Overall adjusted | 481.7 (25.2) | 482.7 (33.2) | 17.1 (1.7) | 17.3 (2.8) | 13.6 (1.7) | 13.4 (2.1) |
| Age group, years |  |  |  |  |  |  |
| 15-19 | 504.3 (65.6) | 504.3 (99.0) | 17.6 (11.5) | 14.8 (14.5) | 14.0 (10.1) | 11.1 (11.9) |
| 20-34 | 487.6 (70.1) | 490.2 (94.7) | 16.0 (9.6) | 12.7 (11.2) | 12.5 (8.1) | 9.6 (9.7) |
| 35-49 | 482.1 (74.1) | 479.9 (97.5) | 16.6 (9.9) | 12.8 (12.6) | 13.5 (9.1) | 9.6 (10.5) |
| 50-65 | 460.4 (73.6) | 460.4 (89.1) | 19.5 (12.5) | 15.4 (14.2) | 16.9 (11.6) | 13.2 (13.7) |
| Sex |  |  |  |  |  |  |
| Male | 502.1 (70.4) | 505.7 (98.4) | 15.8 (9.9) | 12.0 (11.7) | 13.1 (9.0) | 9.7 (10.5) |
| Female | 467.8 (69.9) | 470.1 (91.7) | 18.1 (11.1) | 14.9 (13.5) | 14.3 (9.8) | 10.9 (11.3) |
| Socioeconomic level |  |  |  |  |  |  |
| High | 463.0 (76.2) | 466.8 (96.3) | 17.0 (12.4) | 12.2 (11.7) | 13.7 (11.1) | 9.5 (10.1) |
| Medium | 477.9 (67.9) | 478.3 (94.6) | 16.6 (9.7) | 13.2 (11.7) | 13.3 (8.6) | 9.8 (10.2) |
| Low | 496.7 (70.7) | 495.9 (96.9) | 17.3 (10.4) | 14.3 (13.7) | 14.0 (9.3) | 10.9 (12.1) |
|  | **Venezuela (n=1132)** | |  |  |  |  |
| Overall (g/d) | 273.8 (46.5) | 271.8 (56.4) | 14.5 (9.2) | 10.5 (10.9) | 13.6 (8.4) | 10.0 (10.1) |
| Overall adjusted | 272.2 (5.0) | 270.8 (7.9) | 14.6 (2.0) | 14.1 (2.6) | 13.6 (2.0) | 13.8 (2.9) |
| Age group, years |  |  |  |  |  |  |
| 15-19 | 275.7 (43.8) | 274.0 (54.2) | 17.0 (9.4) | 13.3 (14.4) | 16.7 (8.9) | 13.2 (13.8) |
| 20-34 | 273.0 (45.5) | 270.8 (56.9) | 15.1 (8.7) | 11.4 (11.0) | 14.3 (8.2) | 10.8 (10.6) |
| 35-49 | 273.8 (47.6) | 271.9 (52.9) | 13.8 (9.0) | 9.7 (10.2) | 12.9 (8.2) | 9.0 (9.5) |
| 50-65 | 273.9 (49.3) | 273.7 (59.1) | 12.5 (9.9) | 8.1 (8.2) | 10.5 (7.6) | 7.4 (7.1) |
| Sex |  |  |  |  |  |  |
| Male | 278.0 (47.9) | 276.7 (58.2) | 13.1 (8.1) | 9.5 (9.5) | 12.9 (7.9) | 9.5 (9.5) |
| Female | 269.8 (44.9) | 268.8 (53.6) | 15.9 (9.9) | 11.7 (12.6) | 14.2 (8.8) | 10.4 (10.8) |
| Socioeconomic level |  |  |  |  |  |  |
| High | 260.8 (38.7) | 263.5 (50.4) | 14.9 (9.6) | 10.4 (11.3) | 13.2 (7.7) | 9.4 (10.6) |
| Medium | 274.7 (45.3) | 274.5 (51.6) | 14.1 (9.2) | 10.3 (10.0) | 12.7 (7.6) | 9.7 (9.0) |
| Low | 274.5 (47.2) | 272.2 (58.7) | 14.6 (9.2) | 10.6 (11.1) | 13.8 (8.6) | 10.1 (10.4) |

ELANS Latin American Study of Nutrition and Health, SD standard deviation, IQR interquartile range

^a^ . Estimates obtained from linear regression models with random intercept for study center.

# Online Resource Table 4. Proportion grain foods containing whole grains to total grain foods and the proportion of foods containing >50% whole grains to total grain foods consumed in Latin American countries based on the Latin American Study of Nutrition and Health (ELANS), 2015.

| **Country** | **n** | **Proportion of foods containing whole grain to total grain foods** | | | | **Proportion of foods containing >50% whole grain to total grain foods** | | | |
| --- | --- | --- | --- | --- | --- | --- | --- | --- | --- |
|  |  | **Mean (SD)** | **Mean (SD) adjusted ^a^** | **Median**  **(IQR)** | **Median (IQR) adjusted** | **Mean**  **(SD)** | **Mean (SD) adjusted** | **Median (IQR)** | **Median (IQR) adjusted** |
| ELANS | 9218 | 4.9 (4.2) | 4.6 (0.9) | 3.4 (4.0) | 4.7 (1.4) | 4.1 (3.6) | 3.9 (0.7) | 2.9 (3.4) | 3.8 (0.9) |
| Argentina | 1266 | 5.8 (4.3) | 5.7 (2.0) | 4.3 (4.6) | 5.5 (2.9) | 4.5 (3.8) | 4.4 (1.6) | 3.1 (3.1) | 4.1 (2.4) |
| Brazil | 2000 | 3.6 (4.9) | 3.6 (0.9) | 1.9 (1.2) | 3.6 (1.4) | 2.8 (3.9) | 2.8 (0.6) | 1.6 (0.8) | 2.7 (0.8) |
| Chile | 879 | 4.0 (5.2) | 3.9 (1.2) | 1.9 (1.6) | 3.7 (1.7) | 3.0 (4.1) | 2.9 (0.7) | 1.7 (0.8) | 2.9 (1.1) |
| Colombia | 1230 | 7.2 (4.0) | 7.1 (1.4) | 5.9 (4.5) | 7.1 (1.8) | 6.3 (3.3) | 6.3 (1.0) | 5.3 (3.7) | 6.2 (1.3) |
| Costa Rica | 798 | 5.6 (3.0) | 5.4 (0.8) | 4.7 (3.5) | 5.5 (1.4) | 5.0 (2.7) | 4.9 (0.6) | 4.1 (3.1) | 4.9 (0.9) |
| Ecuador | 800 | 4.3 (3.1) | 4.2 (1.6) | 3.3 (2.7) | 4.0 (2.5) | 3.7 (2.9) | 3.6 (1.5) | 2.8 (2.3) | 3.5 (2.3) |
| Peru | 1113 | 3.6 (2.3) | 3.6 (0.5) | 2.9 (2.6) | 3.7 (0.7) | 2.9 (2.0) | 2.8 (0.4) | 2.2 (2.2) | 2.8 (0.4) |
| Venezuela | 1132 | 5.5 (3.7) | 5.4 (0.8) | 3.7 (4.5) | 5.2 (1.1) | 5.1 (3.4) | 5.0 (0.8) | 3.4 (4.2) | 5.0 (1.2) |

ELANS Latin American Study of Nutrition and Health, SD standard deviation, IQR interquartile range

**^a^** Estimates obtained from linear regression models with random intercept for study center.

# Online Resource Table 5. Coefficients and 95% confidence intervals for the association of sociodemographic variables and the intake of total grain foods, foods containing whole grains, and foods containing >50% whole grains in Latin American countries based on the Latin American Study of Nutrition and Health (ELANS), 2015.

| **Sociodemographic variables** | **Total grain foods** | | **Foods containing whole grains** | | **Foods containing >50% whole grains** | |
| --- | --- | --- | --- | --- | --- | --- |
|  | **β** | **95% CI** | **β** | **95% CI** | **β** | **95% CI** |
|  | **ELANS (n=9218)** | | | | | |
| Age group (Ref. 15-19 y) |  |  |  |  |  |  |
| 20-34 y | -7.52 | -11.17, -3.87 | 0.06 | -0.70, 0.81 | -0.03 | -0.67, 0.61 |
| 35-49 y | -6.68 | -10.49, -2.87 | 0.88 | 0.09, 1.67 | 0.82 | 0.15, 1.50 |
| 50-65 y | -9.82 | -13.87, -5.77 | 3.30 | 2.46, 4.14 | 2.90 | 2.18, 3.61 |
| Sex (Ref. male) |  |  |  |  |  |  |
| Female | -9.92 | -12.22, -7.61 | 4.00 | 3.50, 4.48 | 2.53 | 2.12, 2.94 |
| Socioeconomic level (Ref. high) |  |  |  |  |  |  |
| Medium | 14.66 | 10.45, 18.86 | -1.73 | -2.6, -0.86 | -1.69 | -2.43, -0.95 |
| Low | 24.82 | 20.68, 28.96 | -2.88 | -3.74, -2.02 | -2.23 | -2.96, -1.50 |
|  | **Argentina (n=1266)** | |  |  |  |  |
| Age group (Ref. 15-19 y) |  |  |  |  |  |  |
| 20-34 y | -16.03 | -24.30, -7.77 | 2.46 | 0.63, 4.29 | 1.98 | 0.31, 3.64 |
| 35-49 y | -29.75 | -38.20, -21.29 | 5.15 | 3.27, 7.02 | 4.61 | 2.91, 6.31 |
| 50-65 y | -37.70 | -46.55, -28.85 | 10.81 | 8.85, 12.77 | 8.76 | 6.97, 10.54 |
| Sex (Ref. male) |  |  |  |  |  |  |
| Female | 7.32 | 2.35, 12.29 | 4.16 | 3.06, 5.26 | 3.38 | 2.38, 4.38 |
| Socioeconomic level (Ref. high) |  |  |  |  |  |  |
| Medium | -1.16 | -12.65, 10.32 | -2.27 | -4.81, 0.26 | -2.62 | -4.92, -0.32 |
| Low | 3.61 | -7.88, 15.10 | -3.57 | -6.10, -1.04 | -3.49 | -5.79, -1.19 |
|  | **Brazil (n=2000)** | |  |  |  |  |
| Age group (Ref. 15-19 y) |  |  |  |  |  |  |
| 20-34 y | -2.70 | -11.60, 6.20 | -0.97 | -3.09, 1.15 | -0.75 | -2.44, 0.95 |
| 35-49 y | -3.25 | -12.41, 5.91 | -1.28 | -3.47, 0.90 | -0.92 | -2.67, 0.82 |
| 50-65 y | -8.68 | -18.52, 1.17 | -0.16 | -2.50, 2.18 | 0.46 | -1.41, 2.33 |
| Sex (Ref. male) |  |  |  |  |  |  |
| Female | -10.56 | -15.97, -5.16 | 3.76 | 2.47, 5.04 | 2.18 | 1.16, 3.21 |
| Socioeconomic level (Ref. high) |  |  |  |  |  |  |
| Medium | 19.98 | 9.92, 30.04 | -3.33 | -5.71, -0.95 | -3.22 | -5.11, -1.32 |
| Low | 29.42 | 19.27, 39.58 | -4.73 | -7.13, -2.34 | -3.79 | -5.68, -1.89 |
|  | **Chile (n=879)** |  |  |  |  |  |
| Age group (Ref. 15-19 y) |  |  |  |  |  |  |
| 20-34 | -12.26 | -22.56, -1.96 | -0.55 | -3.80, 2.70 | -0.86 | -3.52, 1.79 |
| 35-49 | -5.04 | -15.68, 5.61 | -1.51 | -4.87, 1.85 | -1.10 | -3.85, 1.64 |
| 50-65 | 0.14 | -10.91, 11.20 | -1.13 | -4.62, 2.35 | -0.64 | -3.49, 2.21 |
| Sex (Ref. male) |  |  |  |  |  |  |
| Female | 7.27 | 0.83, 13.71 | 5.21 | 3.18, 7.25 | 3.12 | 1.46, 4.78 |
| Socioeconomic level (Ref. high) |  |  |  |  |  |  |
| Medium | -4.19 | -15.90, 7.52 | -5.22 | -8.91, -1.52 | -3.40 | -6.42, -0.38 |
| Low | 2.34 | -9.38, 14.05 | -8.76 | -12.46, -5.06 | -5.53 | -8.55, -2.51 |
|  | **Colombia (n=1230)** | |  |  |  |  |
| Age group (Ref. 15-19 y) |  |  |  |  |  |  |
| 20-34 | 3.98 | -4.32, 12.28 | 1.77 | -0.18, 3.73 | 1.62 | 0.03, 3.20 |
| 35-49 | 5.76 | -2.91, 14.42 | 3.96 | 1.92, 5.99 | 3.39 | 1.74, 5.04 |
| 50-65 | 10.43 | 1.65, 19.20 | 7.01 | 4.94, 9.07 | 5.61 | 3.94, 7.29 |
| Sex (Ref. male) |  |  |  |  |  |  |
| Female | -7.81 | -12.81, -2.81 | 4.97 | 3.79, 6.15 | 2.96 | 2.01, 3.92 |
| Socioeconomic level (Ref. high) |  |  |  |  |  |  |
| Medium | 14.45 | 2.88, 26.03 | 0.28 | -2.44, 3.00 | 0.48 | -1.73, 2.68 |
| Low | 23.16 | 12.00, 34.31 | -1.78 | -4.40, 0.84 | -1.22 | -3.35, 0.91 |
|  | **Costa Rica (n=798)** | |  |  |  |  |
| Age group (Ref. 15-19 y) |  |  |  |  |  |  |
| 20-34 | -6.98 | -21.16, 7.21 | 0.77 | -1.23, 2.76 | 0.58 | -1.32, 2.49 |
| 35-49 | 25.27 | 10.41, 40.13 | 2.11 | 0.02, 4.20 | 1.87 | -0.13, 3.86 |
| 50-65 | 35.18 | 19.06, 51.30 | 4.84 | 2.57, 7.11 | 4.47 | 2.31, 6.64 |
| Sex (Ref. male) |  |  |  |  |  |  |
| Female | -23.30 | -32.72, -13.89 | 4.03 | 2.71, 5.36 | 2.10 | 0.83, 3.36 |
| Socioeconomic level (Ref. high) |  |  |  |  |  |  |
| Medium | 14.81 | 0.63, 29.00 | -2.23 | -4.23, -0.24 | -2.45 | -4.35, -0.54 |
| Low | 44.09 | 29.00, 59.18 | -2.36 | -4.48, -0.23 | -2.27 | -4.30, -0.24 |
|  | **Ecuador (n=800)** | |  |  |  |  |
| Age group (Ref. 15-19 y) |  |  |  |  |  |  |
| 20-34 | -16.50 | -27.76, -5.24 | 1.43 | -0.30, 3.17 | 1.52 | -0.02, 3.07 |
| 35-49 | -22.53 | -34.48, -10.58 | 5.15 | 3.31, 7.00 | 4.77 | 3.13, 6.42 |
| 50-65 | -27.51 | -40.84, -14.18 | 11.27 | 9.21, 13.32 | 10.85 | 9.02, 12.69 |
| Sex (Ref. male) |  |  |  |  |  |  |
| Female | -14.83 | -22.45, -7.21 | 5.77 | 4.60, 6.95 | 4.99 | 3.94, 6.04 |
| Socioeconomic level (Ref. high) |  |  |  |  |  |  |
| Medium | 23.55 | 11.29, 35.82 | -0.69 | -2.58, 1.21 | -1.33 | -3.02, 0.36 |
| Low | 34.35 | 22.50, 46.19 | -1.90 | -3.73, -0.06 | -2.08 | -3.72, -0.44 |
|  | **Peru (n=1113)** |  |  |  |  |  |
| Age group (Ref. 15-19 y) |  |  |  |  |  |  |
| 20-34 | -14.18 | -26.16, -2.20 | -1.78 | -3.57, 0.02 | -1.65 | -3.27, -0.03 |
| 35-49 | -18.82 | -31.68, -5.97 | -1.19 | -3.12, 0.73 | -0.63 | -2.36, 1.11 |
| 50-65 | -40.39 | -54.39, -26.38 | 1.55 | -0.55, 3.65 | 2.66 | 0.78, 4.55 |
| Sex (Ref. male) |  |  |  |  |  |  |
| Female | -33.15 | -41.09, -25.22 | 2.25 | 1.06, 3.44 | 1.11 | 0.04, 2.18 |
| Socioeconomic level (Ref. high) |  |  |  |  |  |  |
| Medium | 16.22 | 4.98, 27.46 | -0.54 | -2.22, 1.15 | -0.46 | -1.98, 1.06 |
| Low | 34.67 | 24.02, 45.31 | 0.69 | -0.91, 2.29 | 0.74 | -0.70, 2.18 |
|  | **Venezuela (n=1132)** | |  |  |  |  |
| Age group (Ref. 15-19 y) |  |  |  |  |  |  |
| 20-34 | -2.62 | -10.81, 5.57 | -1.98 | -3.60, -0.35 | -2.42 | -3.89, -0.96 |
| 35-49 | -2.47 | -11.14, 6.20 | -3.23 | -4.95, -1.51 | -3.86 | -5.42, -2.31 |
| 50-65 | -0.90 | -10.32, 8.51 | -4.61 | -6.48, -2.75 | -6.23 | -7.92, -4.54 |
| Sex (Ref. male) |  |  |  |  |  |  |
| Female | -7.92 | -13.17, -2.66 | 3.01 | 1.97, 4.06 | 1.51 | 0.57, 2.46 |
| Socioeconomic level (Ref. high) |  |  |  |  |  |  |
| Medium | 12.43 | -0.58, 25.44 | -0.52 | -3.10, 2.05 | -0.28 | -2.61, 2.05 |
| Low | 11.89 | 0.18, 23.59 | -0.06 | -2.38, 2.26 | 0.76 | -1.34, 2.86 |

CI, confidence intervals, y year, ELANS Latin American Study of Nutrition and Health

# References

1. van der Kamp JW, Poutanen K, Seal CJ, Richardson DP (2014) The HEALTHGRAIN definition of 'whole grain'. Food Nutr Res;58. https://doi.org/10.3402/fnr.v58.22100

2. Ross AB, van der Kamp JW, King R, Lê KA, Mejborn H, Seal CJ, Thielecke F, Healthgrain Forum (2017) Perspective: A Definition for Whole-Grain Food Products-Recommendations from the Healthgrain Forum. Adv Nutr 8:525-531. https://doi.org/10.3945/an.116.014001
